# Heterogeneity in an adeno-associated virus transfection-based production process limits the production efficiency

**DOI:** 10.1038/s41598-025-26261-0

**Published:** 2025-11-04

**Authors:** Brian Ladd, Sofia Tunmats, Torbjörn Gräslund, Olalekan Daramola, Johan Rockberg, Véronique Chotteau

**Affiliations:** 1 AdBIOPRO Competence Centre for Advanced Bioproduction, Stockholm, Sweden; 2 Department of Industrial Biotechnology, KTH Royal Institute of Technology, Stockholm, Sweden; 3 Cell Culture and Fermentation Sciences, Biopharmaceuticals R&D, AstraZeneca, Cambridge, UK; 4 Department of Protein Science, KTH Royal Institute of Technology, Stockholm, Sweden

**Keywords:** AAV, Transient transfection, Transfection heterogeneity, Single-cell transcriptomics, HEK293 cells, Gene therapy, RNA sequencing, Biologics, Gene therapy, Biotechnology, Computational biology and bioinformatics, Data acquisition, Gene ontology

## Abstract

The popularity of rAAV vectors in gene therapy are placing a burden on current production systems. To improve the accessibility of these life changing treatments, increases in production yields and a reduction in the cost-of-goods are needed. Transient transfection is the most common way to introduce rAAV-encoding plasmids to producer cells but it suffers from significant drawbacks such as low and inconsistent yields as well as high cost due to its need for plasmid DNA. This study aims to address the low yield of transient transfection-based rAAV production through advanced methods in process characterization. Adherent and suspension cultures of a HEK293T cell line were triple-transfected for rAAV9 production using polyethylenimine (PEI). Samples were taken at various times post-transfection for analysis with bulk and single-cell transcriptomics. It was revealed that 46% of the cells lacked transcripts of genes from at least one plasmid, indicating that a significant proportion of the cells did not have the genes necessary for rAAV9 production. Among the remaining 54% of the cells expressing genes from all three plasmids, only 8% showed high plasmid gene expression. Flow cytometric analysis of intracellular rAAV9 confirmed these results by showing that only ~ 3% of cells contained assembled rAAV9 capsids. Titre analysis by qPCR of the supernatant and lysate of the producer cells indicated an average culture performance of 10^13^ vg/L. Analysis of the single-cell transcriptomic data showed that a significant proportion of cells that had high plasmid gene expression were in the S-phase. Trajectory inference highlighted that genes involved in the G2-M phase transition, immune response, and protein unfolding were differentially expressed at the branch point between high and low plasmid expression. This study reveals a significant bottleneck in the transient transfection-based production of rAAV. With less than 5% of cells producing rAAV, significant improvements in titres can be achieved if this fraction can be increased. Moreover, regulation of the cell-cycle, inhibition of the immune response, and alleviating protein misfolding all potentially offer the key to enabling these life changing treatments to reach a wider audience.

**Supplementary Information:**

The online version contains supplementary material available at 10.1038/s41598-025-26261-0.

## Introduction

Since the emergence of gene therapy products, recombinant adeno-associated virus (rAAV) has become one of the leading viral vectors. This can be attributed to their unique properties of non-pathogenicity, low immunogenicity, episomal transgene expression, long-term persistency and broad tissue tropism^1–5^. Presently, there are six FDA-approved rAAV-based gene therapies, and over 200 more are under development^6,7^. By 2025, FDA predicts to approve 10 to 20 cell and gene therapies per year^3^. Despite the prevalence of rAAV-based gene therapy, its administration has been limited, partly due to high treatment costs^2,8^. The high prices can be attributed to high dosage requirements^2^, typically in the scale of 10^12^ to 10^15^ vector genomes per systemic dose^6^. This requirement, combined with low manufacturing productivities and high production costs^8^, results in costs per dose in excess of 1 million dollars^9^. The increased adoption of rAAVs places a burden on current manufacturing platforms, and achieving higher productivity is necessary to satisfy the growing clinical and commercial needs.

The most common platform for rAAV manufacture is transient expression in human embryonic kidney 293 (HEK293) cells after triple-plasmid transient transfection^10^. Compared to other methods, transient expression after plasmid transfection is more versatile and forgoes helper viruses, eliminating the risks associated with their use. However, this method faces difficulties during scale-up and is costly due to high plasmid consumption and lack of consistency^7,11,12^. Even with a surplus of plasmids, only 30–80% of cells are successfully transfected^3^. Additionally, plasmid imbalances arising from this method may contribute to inefficient packaging and inconsistent empty-to-full capsid ratios^2^. Hence, titers are generally lower compared to other methods^11^, reaching up to 10^14^ vector genomes per liter^13,14^, requiring several litres of culture for one single dose. Extensive efforts have been made on process development for efficient transfection and transient expression^3,15^. However, the gaps in our biological understanding of rAAV production need to be addressed to overcome the limitations that are hindering the process at a cellular level.

Assembly of rAAVs rely heavily on the host cell machinery, a rational approach to closing the gaps in our knowledge concerning alterations of the host-cell metabolism during AAV production is through the characterization of the host cell transcriptome. Indeed, previous studies have performed bulk RNA sequencing on rAAV production using either HEK293 cells or Sf9 insect cells and identified characteristic features of the producer cells. Virgolini et al. studied the transcriptional changes induced by rAAV production using the Sf9 baculovirus expression vector system. They found an enrichment of transcripts involved in the cell cycle, cell growth, protein folding and amino acid metabolism^16^. Wang et al. compared the transcriptomes of viral-producing and non-producing cells from two different HEK293 cell lines. They found that rAAV production led to an upregulation of innate immune response signalling pathways and stress response pathways of the host cells, as well as late-phase downregulation of fatty acid metabolism and neutral amino acid transport^15^. Chung et al. performed a kinetic study of the transcriptome after transfection at high cell density and at manufacturing scale and found an increased antiviral and inflammatory response, suggesting that cells respond to rAAV production as if it was an infection^17^. Lu et al. studied the cellular response of HEK293 cells towards transient transfection using both transcriptomics and proteomics and found an upregulation in immune response, unfolded protein response and p53 signaling^18^.

Fundamentally, bulk RNA sequencing (RNA seq) studies the average gene expression in a cell population, which conceals the intrinsic cell-to-cell variability that persists even within a homogeneous population^19–21^. Single-cell RNA sequencing (scRNA seq) allows this heterogeneity to be revealed by examining the gene expression at a higher resolution in individual cells^22,23^. As the impact of cellular heterogeneity becomes more recognized and as advancements improve the accuracy and cost-effectiveness of this technology^24^, more studies have adopted scRNA seq for exploring the gene expression of cells in various contexts. Most of these studies are within immunology and oncology, revealing tumor heterogeneity and distinguishing immune cell types in clonally distinct cells^20^. However, one study has applied scRNA seq to examine rAAV production using the insect-cell baculovirus expression vector system in Sf9 insect cells^25^.  Virgolini et al. found a progressive increase in heterogeneity associated with the expression of viral and viral vector genes, as well as alterations in protein folding and translation, metabolic processes and stress response due to infection.

This study presents the first application of scRNA seq to adherent and suspension cultures of HEK293 cells during transient transfection-based rAAV production, which uncovered cell-to-cell variability and potentially its underlying causes. Distinct groups were identified among the cells producing rAAVs and the trajectory of their development was examined. Using this approach, a significant limitation in production was identified and strategies for supporting rAAV production are proposed.

##  Materials and methods

### Cell culture

HEK293T/17 ATCC-CRL-11,268 cells (see Ladd et al.^26^ were grown in GIBCO FreeStyle HEK293 Expression medium (Thermo Fisher Scientific, Waltham, USA) as either adherent cultures in Corning CellBIND static tissue culture T-25 flasks (Corning Inc., New York, USA) or suspension cultures in 125 mL shake flasks. All cultures were maintained in a Minitron incubator shaker set to 37 °C and 5% CO2.

### rAAV production

Prior to transfection, the adherent and suspension cultures reached cell densities of 2 × 10^6^ cells mL^− 1^. Equal concentrations of the transfection agent PEIpro^®^ (Polyplus, France) diluted in the cell culture medium and a DNA mixture containing a 2:1:1 mass ratio of pHelper, pRepCap (rAAV9), and green fluorescent protein (GFP) plasmids (Cobra Biologics, Charles River, UK) were combined. The PEIpro-DNA mixture was incubated for 7 min and then added to the cultures. The adherent and suspension cultures were maintained for up to 48 h post transfection (hpt). Samples were collected at 0, 12, 24 and 48 hpt and cryopreserved in 7.5% dimethylsulfoxide (DMSO).

### Analytics

#### Cell density and viability

Cell density and viability of the samples were determined with the NORMA XS automated cell counter and cell viability analyzer (IPRASENSE, Clapiers, France), which is based on holographic imaging.

#### GFP expression

Samples taken from the cultures were centrifuged at 180 g for 5 min and resuspended in phosphate-buffered saline (PBS). The resuspended cells were analyzed using the Guava easyCyte benchtop flow cytometer (Cytek Biosciences, Fremont, USA). The forward and side scattering were used to gate single cells in the InCyte software Version 4.5 (Cytek Biosciences, Fremont, USA). GFP fluorescence was measured with a 488 nm excitation laser and a combination of a 512/18 nm and a 575/25 nm detector.

#### rAAV genome titers

rAAV genome titer quantification was performed using the AAVpro Titration Kit (for Real Time PCR) Ver.2 (cat#6233) (Takara Bio, Kusatsu, Japan). The samples were prepared following the associated protocol and using the provided kit components. The adherent samples were first washed with Ca^2+^- and Mg^2+^-free PBS, then incubated with TrypLE (Thermo Scientific, Waltham, USA) at 37 °C for 5 min. Supernatant samples were obtained by centrifuging at 1000 g for 5 min. Lysate samples were prepared through 3x freeze thaw cycles in a lysis buffer at -80 °C and centrifuged at 1000 g for 5 min. The lysis buffer containing 150 mM NaCl, 50 mM Tris-HCl and 2 mM MgCl_2_ was adjusted to a pH of 8.5 using NaOH. The lysate and supernatant samples were separately treated with DNAse for 1 h at 37 °C to digest free DNA and subsequently heated to 95 °C for 30 min to inactivate the DNAse. The lysate and supernatant samples were treated with lysis buffer for 20 min to break down the rAAV9 capsids. Finally, the samples were diluted 50-fold to use as a template for real-time qPCR. TB Green *Premix Ex Taq* II and the AAV2 ITR Forward and Reverse Titer Primers were combined with either the lysis template, supernatant template or the positive control provided in the kit prior to executing real-time qPCR analysis using the Bio-Rad CFX96 qPCR System (Bio-Rad, Hercules, USA). The program included: an initial denaturation at 95 °C for 10 min, a 2-step PCR comprising 35 cycles at 95 °C for 5 s and at 60 °C for 30 s for FAM fluorescence detection and a melt curve analysis. The standard curve was generated through a serial dilution of the positive control to facilitate quantification.

### Intracellular rAAV assembled capsid distribution

The distribution of rAAV particles containing correctly assembled capsids in the suspension sample at 48 hpt was investigated using the biotinylated affinity ligand CaptureSelect Biotin Anti-AAV9 conjugate (Thermo Fisher Scientific, Waltham, USA) which specifically binds to assembled capsids. At 48 hpt, cells were first washed with PBS, then fixated using 4% paraformaldehyde in PBS for 15 min. The fixated cells were washed with PBS and centrifuged at 400 g for 5 min three times to remove the fixative. The fixated cells were subsequently permeabilized with 0.1% Triton-X in PBST (PBS with 0.1% Tween 20) for 7 min and centrifuged at 400 g for 5 min. The permeabilized cells were incubated at room temperature with 0.1 ng/µL of the biotin anti-AAV9 conjugate for 1 h. This was followed by incubation at room temperature with 0.5 ng/µL Streptavidin, Alexa Fluor 647 Dye (Thermo Fisher Scientific, Waltham, USA) for 1 h. After incubation, the cells were washed with PBST, centrifuged at 400 g for 5 min and then resuspended in PBST. The samples were analyzed with the Guava easyCyte benchtop flow cytometer (Cytek Biosciences, Fremont, USA) using a 642 nm excitation laser and a 661/15 nm detector. The gating was performed in the InCyte software Version 4.5 (Cytek Biosciences, Fremont, USA) using the forward and side scattering. Untransfected cells were used as a negative control.

### Single-cell RNA sequencing

#### Sample preparation for sequencing

The samples for scRNA seq were thawed in a water bath at 37 °C prior to multiplexing with the Single cell Multiplexing Kit (Cat. No. 633781) (BD Biosciences, Franklin Lakes, USA) according to the manufacturer’s instructions (Doc ID: 210970). The isolation of single-cells, subsequent capture of the RNA, and then cDNA synthesis was performed with the BD Rhapsody Express (BD Biosciences, Franklin Lakes, USA) according to the manufacturer’s protocol (Doc ID: 210967) using one cartridge. A target of 5 000 cells per sample was used, which resulted in a total of 20 000 cells per cartridge. The Whole Transcriptome Analysis (WTA) Amplification Kit (Cat num: 633801) (BD Biosciences, Franklin Lakes, USA) was used to prepare the libraries from the cDNA containing BD Rhapsody beads according to the manufacturer’s protocol (Doc ID: 23-21712-00). The library quality and fragment length were checked with capillary gel electrophoresis on an Agilent 2100 Bioanalyzer (Agilent Technologies, Santa Clara, USA). The High Sensitivity DNA kit (Cat. No. 5067 − 4626) (Agilent Technologies, Santa Clara, USA) was used according to the manufacturer’s protocol. Paired-end mRNA sequencing and adapter demultiplexing using the Illumina NovaSeq 6000 platform (Illumina, Inc., USA) was performed by a third party (Novogene Co., LTD, China).

#### Reference transcriptome for alignment

Whole plasmid sequencing of the plasmids was performed by a third party (Eurofins, Germany) through real-time long-read sequencing using Oxford Nanopore technology. The sequences for the human adenovirus 5 (Ad5) genome (AC_000008.1) and the simian virus 40 large T antigen (SV40 LT) antigen gene (AY531219.1) were acquired from the National Center to Biotechnology Information (NCBI)^27^. The plasmid sequences, Ad5 genome and SV40 LT gene were converted into transcripts using the program created by Pertea et al. called GFFRead^28^. This program used the obtained FASTA and general feature formats (GFF) files to convert the genes into transcripts. The transcripts for the plasmid sequences, Ad5 genome and SV40 LT gene combined with the human reference transcriptome from the GRCh38.p14 assembly (GCF_000001405.40) in NCBI were used as references for the alignment.

#### Sequence alignment

Sequence alignment to the references was performed in the cloud-based bioinformatics platform Seven Bridges (Seven Bridges Genomics Inc., USA). From this platform, the BD Rhapsody Sequence Analysis Pipeline version 2.0 utilizing the STAR alignment algorithm^29^. The output FASTQ files obtained from the sequencing and the reads are filtered by quality, demultiplexed and aligned to the references. Unique molecular identifiers (UMI) were quantified per gene per cell. Finally, UMI correction and cell label filtering were performed to obtain the file containing the molecule counts per cell, the binary alignment map (BAM) and the sequencing metrics.

### Data analysis

#### Processing and clustering

The output file from the alignment was processed using the package Seurat version 5.1.0^30^, in R version 4.3.1. Multiplets, undetermined cells, low quality cells (> 15% mitochondrial features and < 3000 gene count) were removed from the dataset. The dataset was split into two subsets of either adherent or suspension cells. The counts within these subsets were log normalized and features were linearly scaled using the functions NormalizeData() and ScaleData() with default parameters. PCA dimensionality reduction was performed with 50 principal components. An equivalent number of dimensions was used to construct the nearest-neighbor graph with the function findNeighbors(). The clustering was performed with the function findClusters(). The resolution for the clustering was determined using the framework by Patterson-Cross et al. called chooseR^31^, which chooses the most robust parameter through bootstrapped iterative clustering. A significance analysis of the number of clusters was performed using the package scSHC version 0.1.0^32^, confirming that the number of principal components and the resolution were optimal for the subsets.

#### Module scores

The module scores for the plasmid gene expression were calculated using the AddModuleScore() function in Seurat version 5.1.0^30^ with default parameters.

#### Trajectory inference

Trajectory inference was performed on the adherent and suspension subsets separately with Slingshot version 2.10.0 to follow the progression of cells between different cellular states over an underlying temporal variable or pseudotime^33^. Determination of the starting cluster was guided by the plasmid expression. A module score was calculated for the combined expression of all three plasmid genes, and the cluster with the lowest expression was defined as the starting cluster. Using default parameters in Slingshot, lineages were determined for the remaining clusters. Each lineage branching from the starting cluster represents cells transitioning into different states. From these lineages, principal curves were fitted through the data. Each principal curve is a smooth non-linear representation of the lineage. The cell-level weights and pseudotimes were individually extracted for the lineages for further analysis.

####  Trajectory-based differential expression and overrepresentation analysis

Trajectory-based differential expression analysis was individually performed on the lineages using the packages tradeSeq version 1.16.0^34^ and SingleCellExperiment version 1.24.0^35^. A negative binomial general additive model (NB-GAM) was used to model gene expression of each gene as smooth nonlinear functions of pseudotime. The optimal number of knots, at which the smooth nonlinear functions are joined together, was determined to be 6 using the function evaluateK(). The cell-level weights and pseudotimes were used as input values, while keeping all other parameters default. To determine the genes that are differentially expressed between the starting and end points of the lineage, the function startVsEndTest() was individually used on the gene NB-GAMs for each lineage. Genes with a log2 fold change > 2 and a p-value < 0.05 were clustered based on similarities in expression pattern over 100 points along the trajectory using the function clusterExpressionPatterns(). The function RSEC() from the package clusterExperiment version 2.22.0^36^ was then used to consolidate the clusters into a final consensus cluster. The merging cut-off was set to 0.9 while keeping all other parameters default. The final consensus clusters were ordered based on gene expression over pseudotime. Overrepresentation analysis on each of the final consensus clusters was performed using the packages clusterProfiler version 4.10.1^37^ and enrichR version 3.2^38^ to determine the Gene Ontology (GO) biological processes associated with each final consensus cluster. An adjusted p-value threshold of < 0.0001 was applied to show only the most significant terms.

### Comparison of single-cell and bulk plasmid gene expression

#### Sample preparation for bulk RNA sequencing

A second experiment was conducted to produce samples for bulk RNA seq using the same methods described in Sect. 2.1, 2.2 and 2.3. Four replicates were prepared and sampled at 0, 12, 24 and 48 hpt. Samples were cryopreserved in 7.5% DMSO prior to sequencing. Library preparation and paired-end mRNA sequencing was performed on the cryopreserved samples by an external party (Novogene Co., LTD, China) using the Illumina NovaSeq 6000 platform. The external party performed quality control and data filtering by removing reads containing adapters, reads with > 10% indeterminable (N) bases, and reads with low quality (Q score ≤ 5) bases comprising > 50% of the total bases.

#### Alignment and quality control

Quality control of the acquired bulk RNA sequencing data was performed prior to data processing using a tool created by Andrews et al. called FastQC^39^ combined with the tool made by Ewels et al. called MultiQC^40^. Alignment and quantification was performed using a program developed by Bray et al. called Kallisto^41^. The reference index used for pseudoalignment and quantification was created in Kallisto with the same reference transcripts and transcriptomes used in 2.5.2. The output file of Kallisto containing the estimated counts for each transcript was used for data analysis. Quality control of the pseudoalignment was performed using MultiQC^40^.

#### Data processing and analysis

The package DESeq2^42^ was employed to normalize the raw counts of the bulk RNA seq experiment using a negative binomial model. For comparison with the scRNA seq experiment, the raw counts of the plasmid genes from the bulk RNA seq experiment were normalized to their respective values at 48 hpt. These values were then compared to pseudo bulked raw counts from single-cell samples. Pseudo bulking was achieved using the function AggregateExpression from the package Seurat version 5.1.0^30^. The raw counts from all the cells were summed for each gene and the summed counts were normalized to their respective values at 48 hpt.

## Results

### Transient rAAV production through transient triple-plasmid transfection

**Fig. 1:**
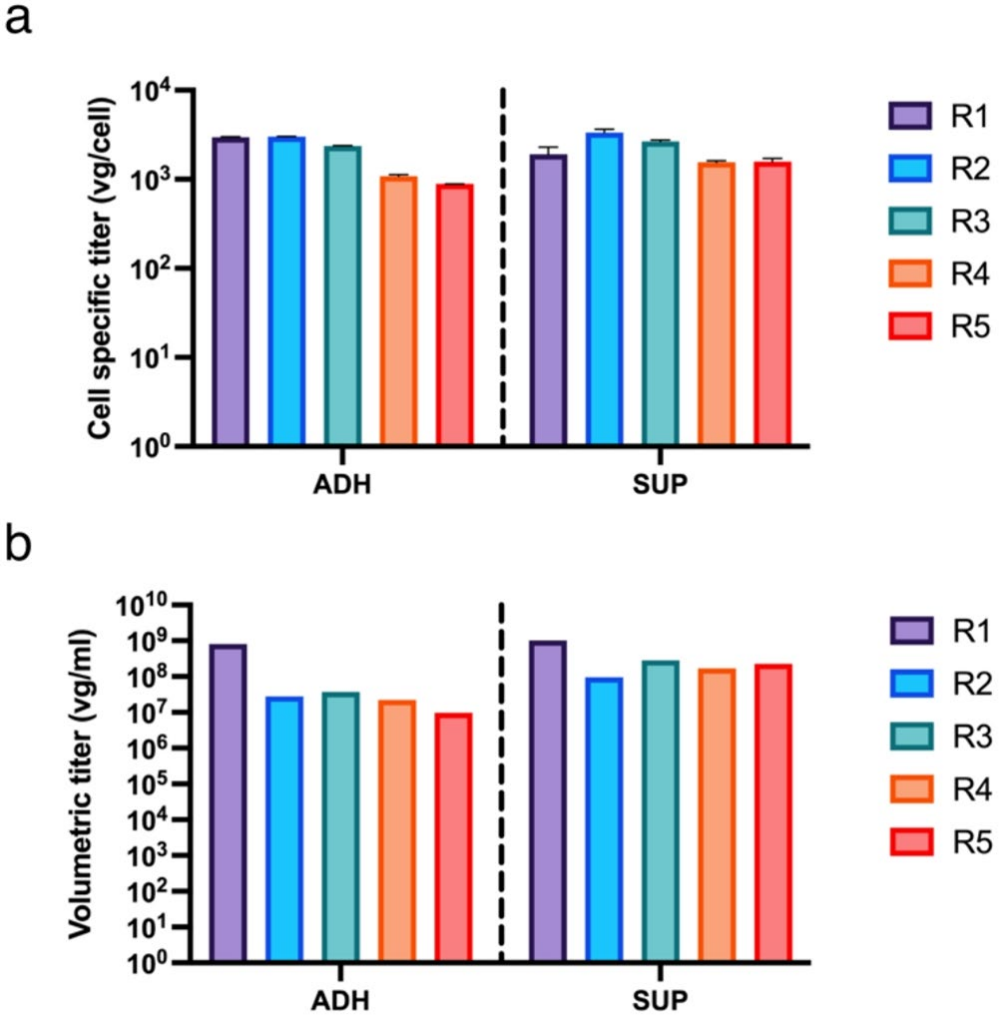
(**a**) Cell specific titer in the lysate and (**b**) volumetric titer in the supernatant measured through qPCR for adherent (ADH) and suspension (SUP) samples at 48 hpt. R1 to R5 corresponds to five replicates of the same experiment. One replicate (R1) was used for the single-cell RNA sequencing experiment and four replicates (R2-R5) were used for the bulk RNA sequencing experiment.

Two HEK293 cell clones, growing either adherent or in suspension, were transfected with three plasmids for the production of rAAV9. Analysis of the cell lysates for rAAV titers revealed broadly similar values for the adherent and suspension cultures, around 2000 vg/cell, shown in Fig. 1. However, in the supernatants, the suspension cultures produced slightly more with 10^8^ vg/mL compared to the 10^7^ vg/mL of the adherent cells. There were slight variations among replicates, particularly for R4 and R5 in comparison with the other replicates.

### Single-cell and bulk data quality control and processing

Next, the transcriptomes of the cells were determined, both for single cells (R1) and bulk (R2-R5). The quality filtering, alignment, UMI correction and cell label filtering for the scRNA seq samples resulted in an average of 3.8 × 10^3^ reads per cell for all four samples, adherent growth and suspension growth at 12 and 48 hpt, as shown in Table S1. The resulting number of cells after filtering is shown in Table S1. The bulk RNA seq data had an average of 2.6 × 10^7^ normalized counts per sample after quality filtering, alignment and normalization, given in Table S2.

### Correlation of single-cell plasmid gene expression to bulk

**Fig. 2:**
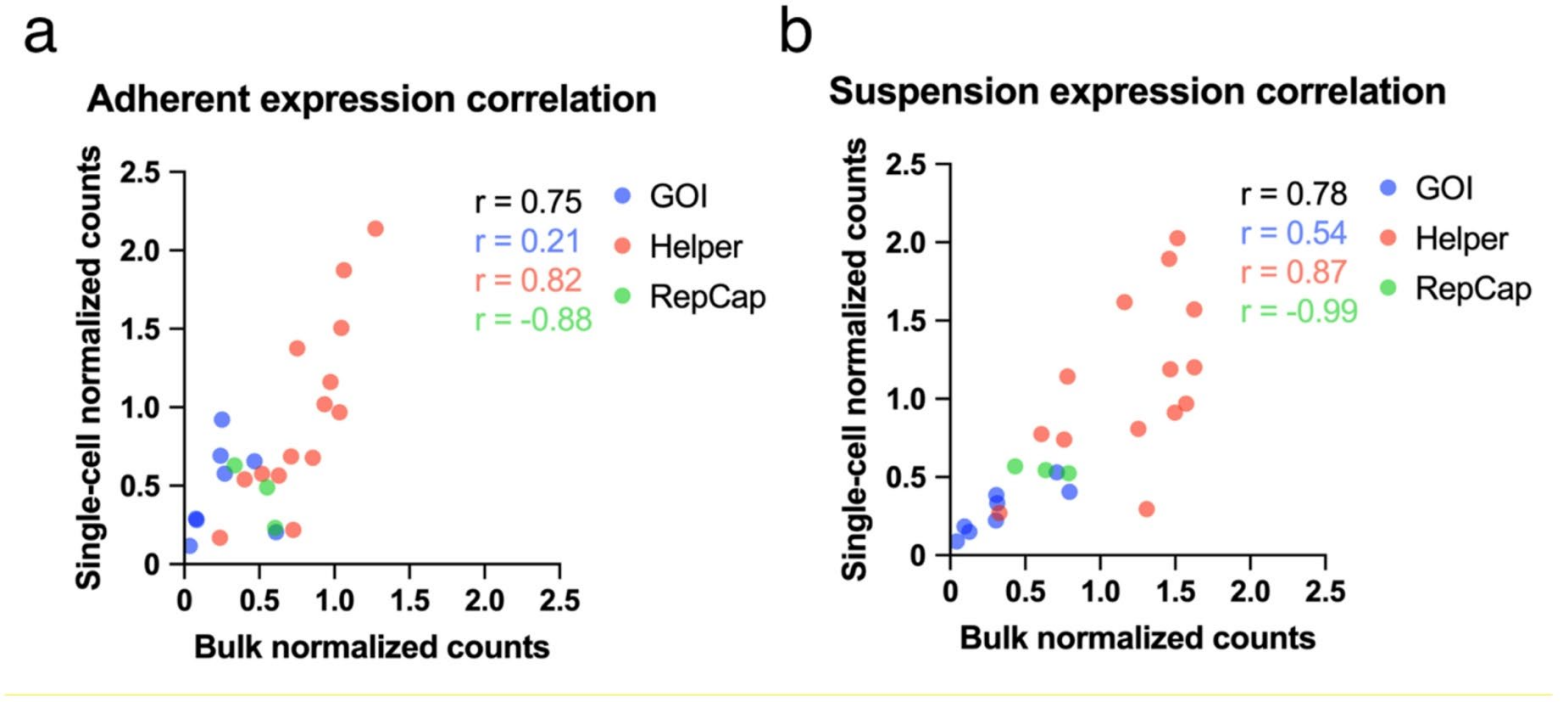
Correlation between bulk and single-cell normalized counts of all plasmid genes obtained during RNA sequencing expressed in the adherent (**a**) and suspension (**b**) cells at 12 hpt. The genes are grouped by plasmid of origin: pGOI (blue), pHelper (red), pRepCap (green). The Pearson correlation coefficients (r) are displayed for each group as well as the combined plasmid expression (black).

To confirm the reproducibility of the plasmid gene expression observed in the single-cell RNA seq experiment, the single-cell raw counts were pseudo bulked then compared to the bulk RNA seq counts. The raw counts from each plasmid gene from 12 hpt of both experiments were normalized to the corresponding gene expression at 48 hpt, shown in Figure S1. The relationship of expression of all of the plasmid genes between the bulk and scRNA seq experiments is shown in Fig. 2. Pearson correlation coefficients (r) were calculated for the three plasmids separately. The genes of the pRepCap plasmid had a high correlation between the bulk and scRNA seq experiments. The genes of the pHelper and pGOI plasmids had, however, a less convincing correlation. This was mostly driven by the few genes in the pRepCap plasmids and by a few outliers in the pGOI plasmid, particularly for the adherent samples. An overall r value of > 0.7 was found for all plasmids for both adherent and suspension samples, demonstrating a good overall correlation of plasmid expression between the two experiments.

### Heterogeneity observed in the plasmid gene expression

**Fig. 3:**
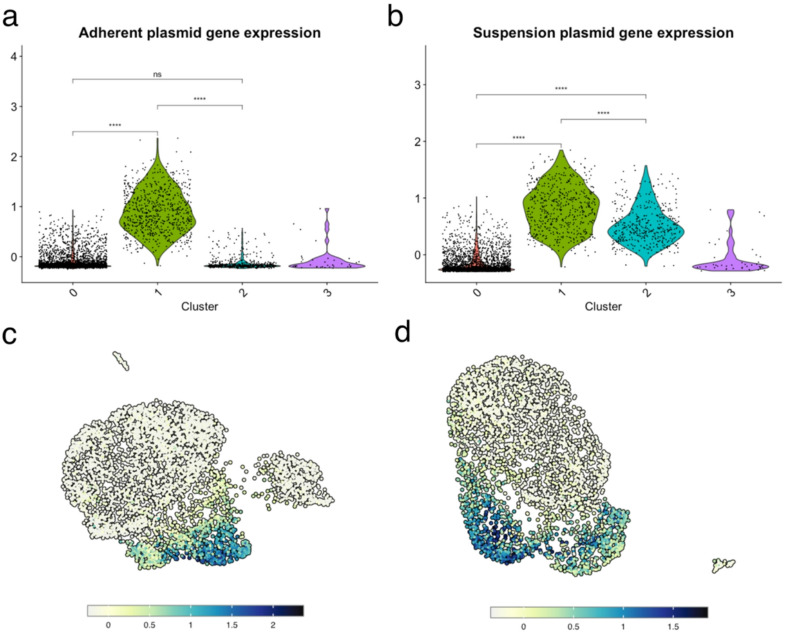
Violin plot of adherent (**a**) and suspension (**b**) subsets of the single-cell RNA sequencing data showing the module score for plasmid gene expression of each cluster. UMAP plot highlighting the plasmid gene expression score in the adherent (**c**) and suspension (**d**) subsets.

Individual clustering of the two subsets (adherent and suspension) resulted in four distinct clusters in both cases, seen in Figure S2. Cluster 3 for both subsets was found to have high mitochondrial gene expression, potentially indicating dead or dying cells, and was therefore excluded from the rest of the analysis. Using the plasmid genes as a signature, we calculated a module score and classified cells with scores at or above the 95th percentile as having high plasmid expression, the cutoffs of which are shown in Figure S3. The highest plasmid gene expression was observed in cluster 1 for both subsets, shown in Fig. 3. Clusters 0 and 3 had comparably low plasmid gene expression for both subsets and the plasmid gene expression of cluster 2 differed between the subsets. Some cells had no expression of any plasmid genes (2.3% of adherent and 1.4% of the suspension subsets) and were mainly found in cluster 0, as shown in Figure S4. Other cells expressed all plasmid genes (48% of adherent and 63.9% of the suspension subsets) and were ubiquitous in all clusters, also exhibited by Figure S4.

Thus, it was concluded that cluster 1 contained cells with high plasmid gene expression and cluster 2 contained cells with low plasmid gene expression. Cluster 0 had low or no plasmid gene expression, likely comprising of “progenitor cells” to the other clusters.

**Fig. 4:**
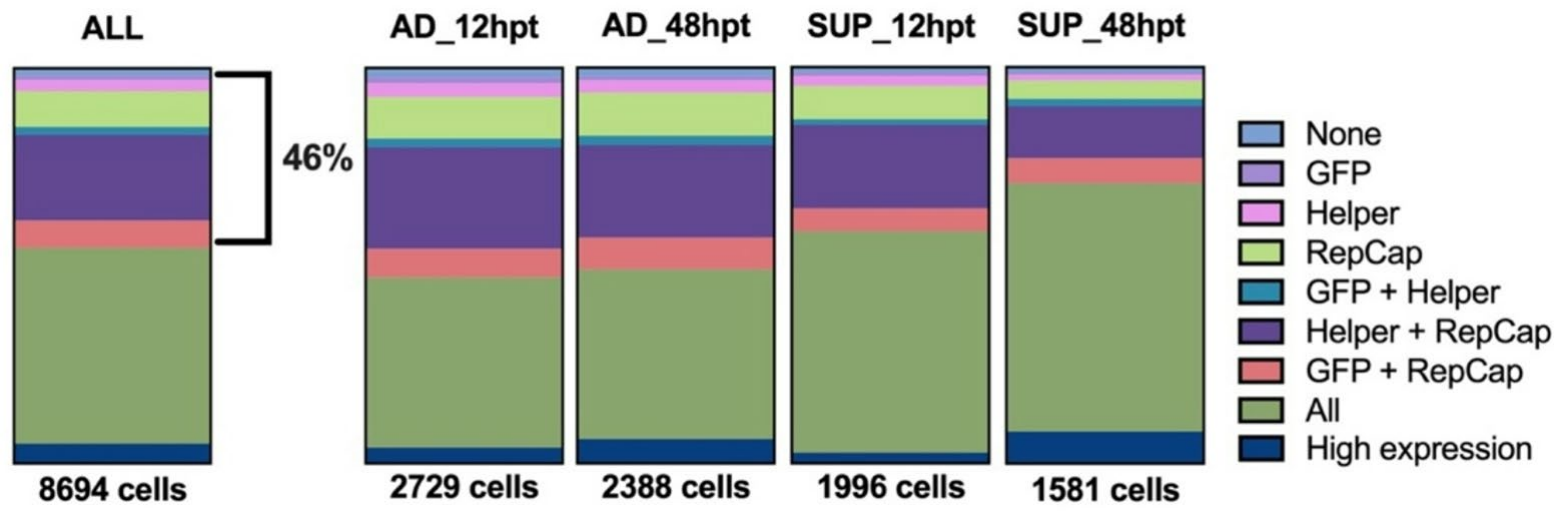
Plasmid gene expression of the cells from the single-cell RNA sequencing experiment. Plasmid expression is attributed to a cell if at least 1 transcript of any gene from that plasmid exists in that cell. Cells expressing all plasmid genes (olive green) and cells with high plasmid gene expression (navy blue) are also shown. To the left, the plasmid gene expression of all samples combined is displayed.

A large percentage of the cells (46%) were missing gene expression of at least one plasmid, as shown in Fig. 4. The percentage of cells missing plasmid gene expression decreased over time and was generally lower in samples taken at 48 hpt, especially for the suspension subset. This indicated that not all cells are expressing genes to a detectable level from all plasmids at 12 hpt. A marginal increase over time was also observed for cells with high plasmid gene expression for both subsets. The adherent and suspension samples had comparable levels of high plasmid gene expression, 5% and 8%, respectively.

**Fig. 5:**
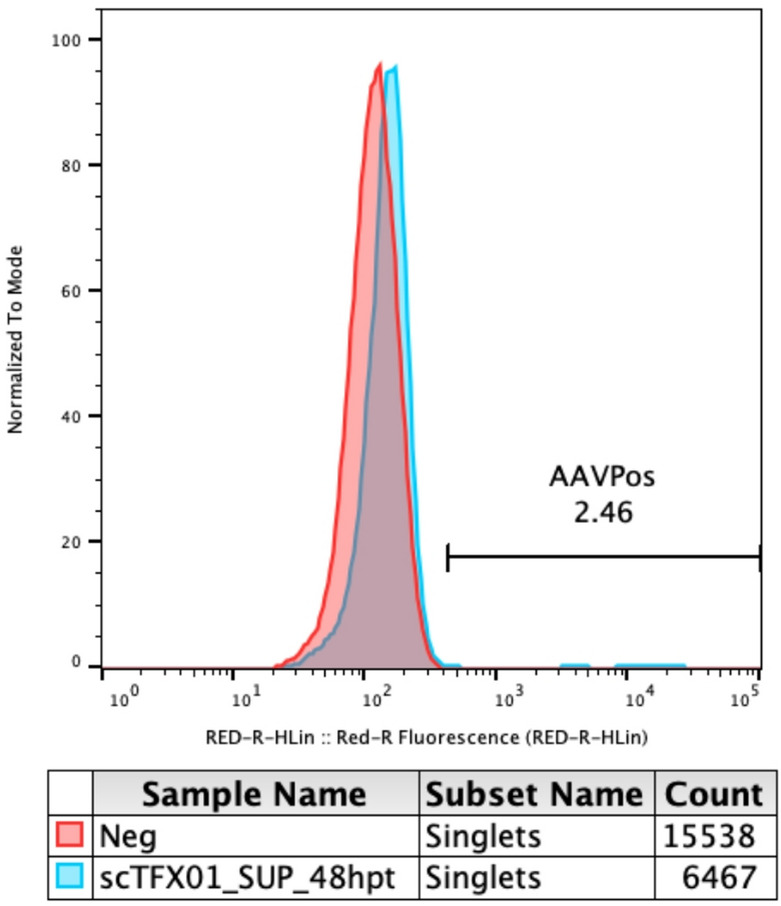
Flow cytometry histogram with suspension cells at 48 hpt stained for intracellular assembled rAAV capsids and filtered from debris and multiplets. The negative control comprises stained untransfected cells. The positive gate was set based on the negative control.

To further investigate this observed heterogeneity in plasmid gene expression, intracellular rAAV capsids were stained using a nanobody specific to assembled rAAV9 capsids. This revealed that only a small percentage of cells (2.5%) from the 48 hpt suspension subset had intracellular assembled capsids to any large extent, shown in Fig. 5.

### Different cellular trajectories determine the plasmid gene expression

**Fig. 6:**
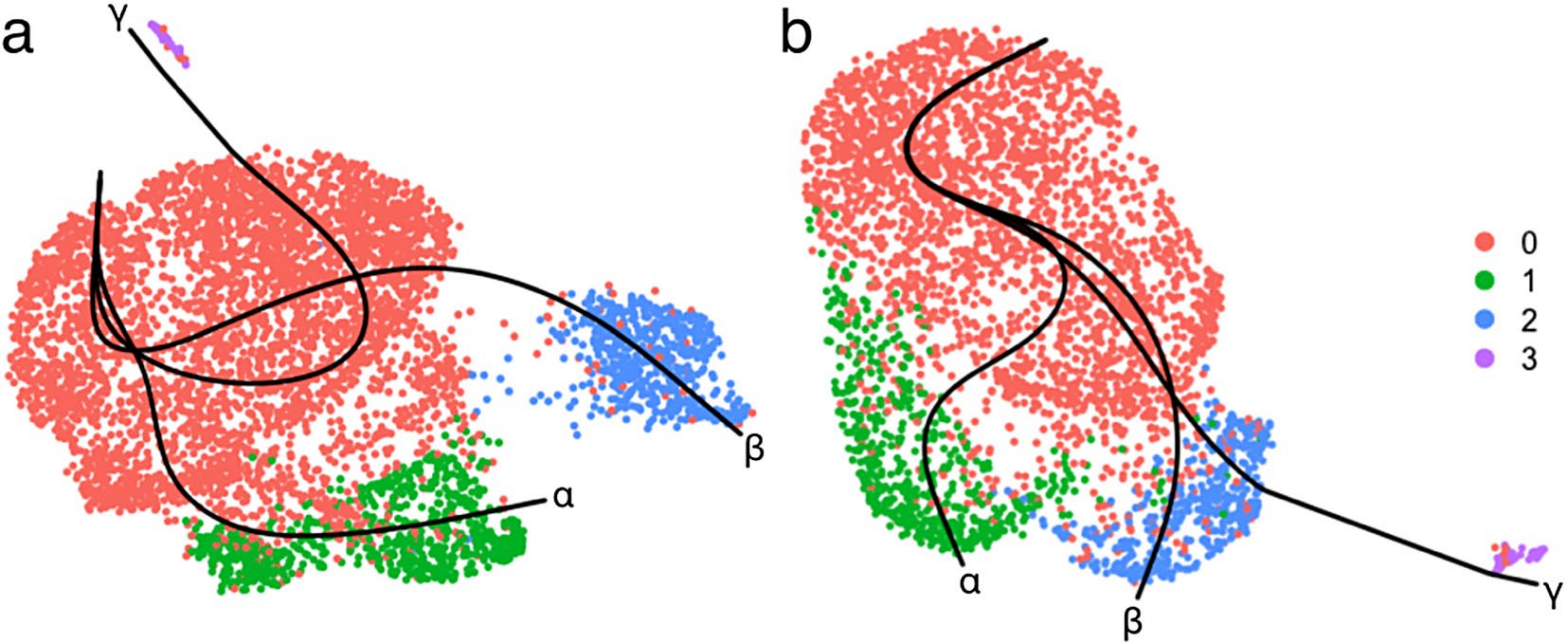
UMAP plot with the three lineages (α, β, γ) for the adherent (**a**) and suspension (**b**) subsets of the single-cell RNA sequencing data generated from the trajectory inference. The cells are colored by cluster: 0 (red), 1 (green), 2 (blue), 3 (purple).

Trajectory inference was performed on the subsets to reveal the progression of cells toward different states. Using cluster 0 as the starting cluster, three lineages (α, β, γ) were identified, Fig. 6. The distance traveled from the beginning of the lineage is denoted as pseudotime. These lineages show that the progenitor cells, cluster 0, could either progress into cells with high plasmid gene expression, cluster 1, following the α lineage or into cells with low plasmid gene expression, cluster 2, following the β lineage. The γ lineage leading to cluster 3 was excluded from the analysis.

**Fig. 7:**
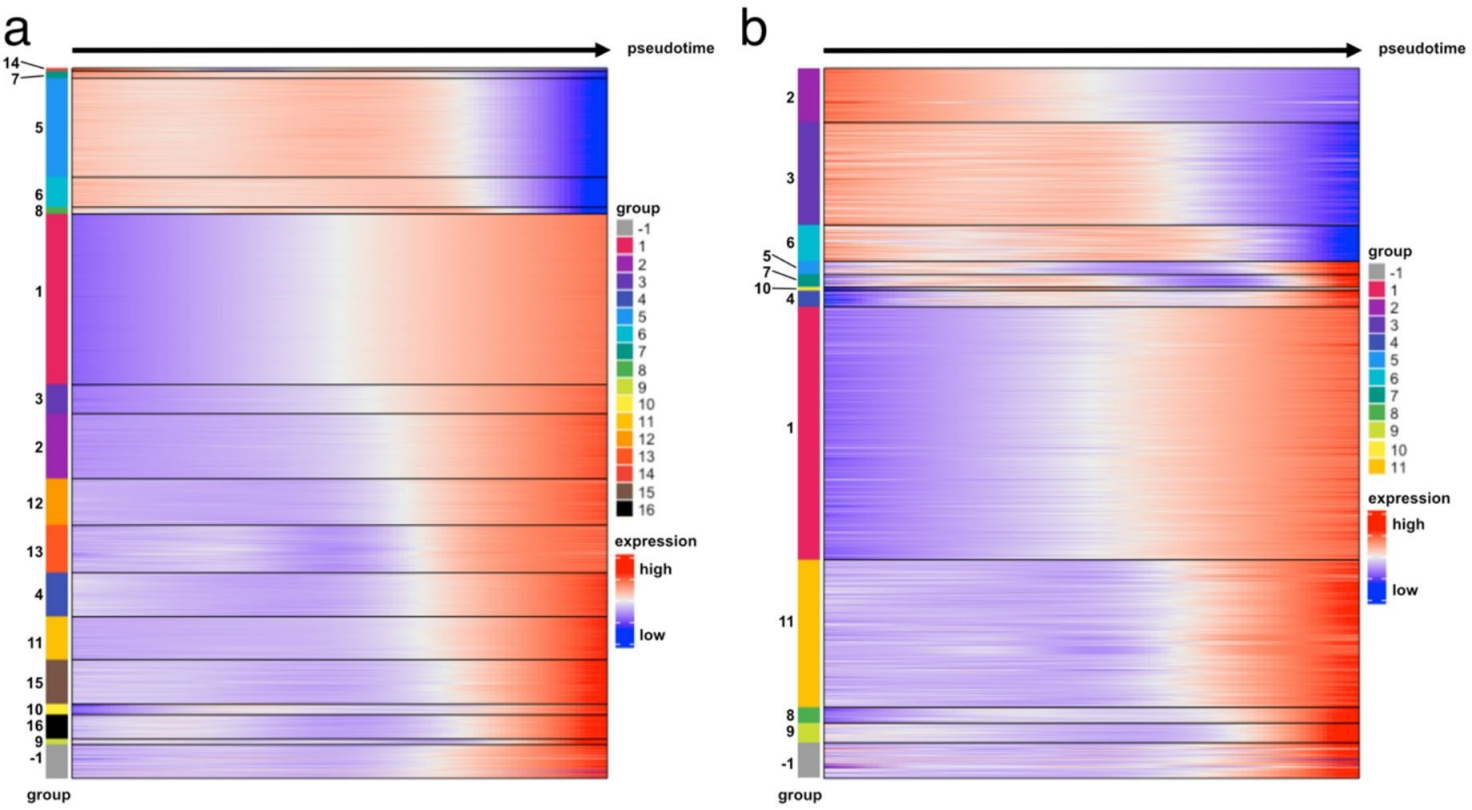
Heatmap plot of the relative expression levels (log2 normalized z-score) of differentially expressed genes associated with the trajectory of suspension lineages towards low (**a**) or high (**b**) plasmid gene expression. The genes are grouped based on expression pattern over pseudotime. The group labelled − 1 shows genes that were not successfully grouped.

**Fig. 8:**
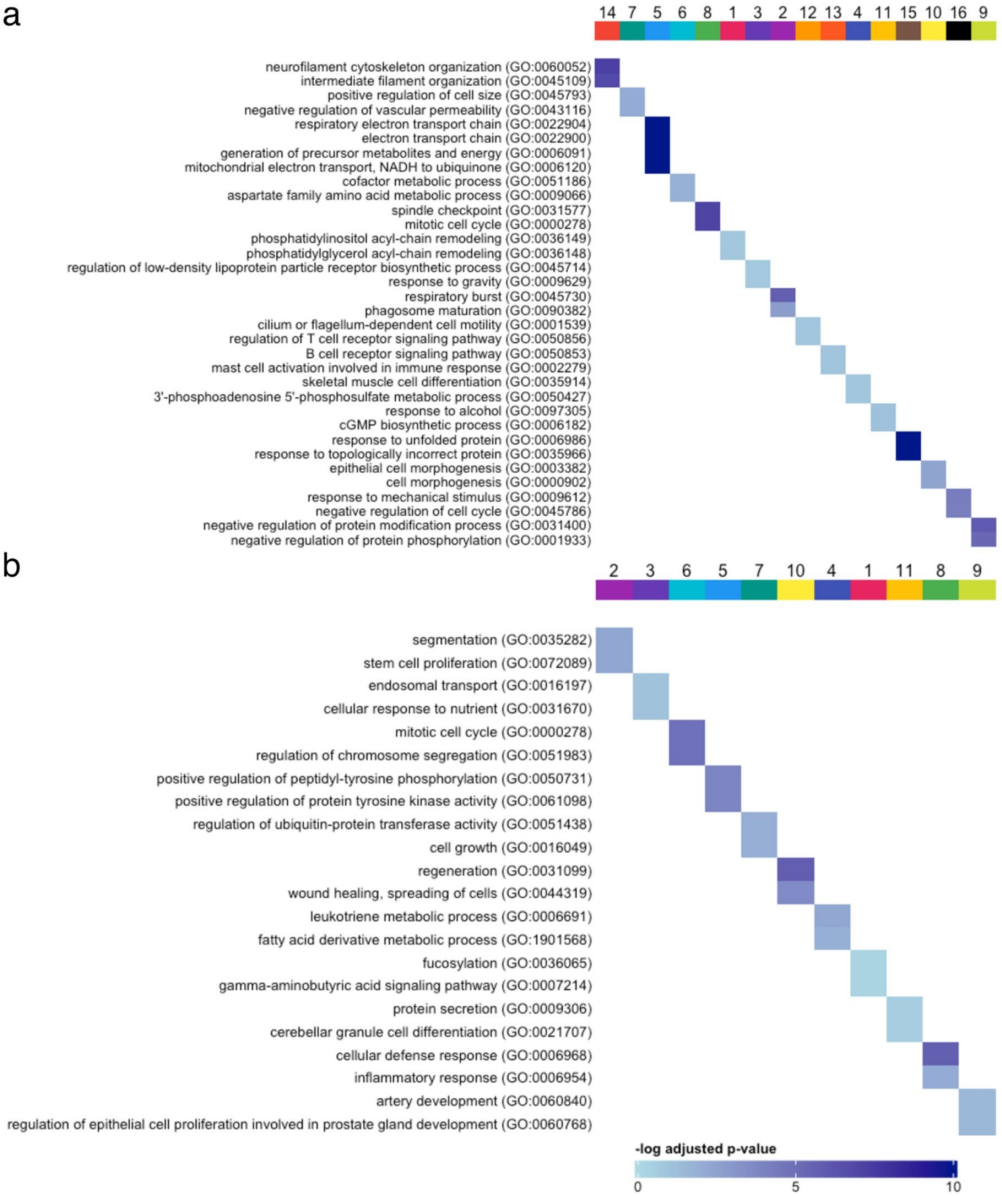
Results from the overrepresentation analysis with Gene Ontology biological processes of groups of differentially expressed genes associated with the suspension lineages towards low (**a**) or high (**b**) plasmid gene expression. The genes are grouped based on expression pattern over pseudotime.

Differential expression and overrepresentation analysis on the lineages were performed to identify factors that determined cellular fate. For each of the lineages, genes were grouped based on their expression over pseudotime, as shown in Figs. 7 and S5 for suspension and adherent cells. The number of groups identified varied between 9 and 16 for the lineages of the adherent and suspension subsets. Some of the genes could not be merged to any of the groups and thus formed their own grouping of unmatched genes (denoted − 1). Notably, some groups had cycles of gene expression over pseudotime. Significant GO terms were determined for the gene groups and ordered based on their expression over pseudotime, shown in Figs. 8 and S6. The full list of significant GO terms can be found in Tables S3 – S6.

## Discussion

Transient transfection for the production of rAAVs suffers from many limitations^13^, some of which may be caused by cellular heterogeneity^43^. Heterogeneity is intrinsic to most cellular systems and can exist at different levels, from genomic to proteomic, and the extent of heterogeneity between these levels may vary^20,21^. A recent study investigated the cell-to-cell variability in a limited population of non-transfected adherent HEK293FT cells using scRNA seq and found only low levels of biological variance, which were primarily due to that the cells were at different stages in the cell cycle^44^. However, after viral infection and propagation, high heterogeneity of gene expression has been observed^45,46^. Single-cell transcriptomics could be a powerful tool for revealing the extent and cause of heterogeneity during transient transfection-based rAAV production^43^. Using single-cell transcriptomics, we were able to identify subpopulations of cells exhibiting high or low rAAV plasmid gene expression and determine the characteristic features for these subpopulations.

Samples taken at 12 and 48 hpt were used for scRNA seq to reflect the early and later stages of transfection, as viral replication begins at 12 hpt and the majority of virions are produced after 24 hpt^47,48^. The normalized expressions of plasmid genes in pseudo bulked single-cell samples and bulk samples generally followed similar dynamics. However, genes of the pGOI plasmid tended to be higher at 12 hpt in single-cell samples than in bulk. Correlation analysis between single-cell and bulk plasmid gene expression resulted in a good correlation for the genes of the pHelper plasmid in both adherent and suspension cell samples. Only three genes were available for comparison for the gene of the pRepCap plasmid, indicating that the observed high negative correlation may lack substantial significance. On the other hand, a low r value for the genes of the pGOI plasmid was obtained, in particular for the samples of adherent cells. A more detailed analysis of the data revealed a significant enhancement of the correlation of genes from the pGOI plasmid for both adherent and suspension cell samples through the omittance of the GOI ORI encoding sequence. In general, a high correlation was found between bulk and single-cell plasmid gene expression. Importantly, the mRNA expression may not reflect the corresponding protein expression for every gene in a cell^49,50^. However, it can give indications of changes in cellular response to different stimuli as well as changes in expression level of individual proteins over time.

Clustering of the scRNA seq data was found to be greatly influenced by the plasmid gene expression, indicating a separation of subpopulations of cells with high or low plasmid gene expression. Both the adherent and suspension subsets acquired a positively skewed distribution of the plasmid gene expression module score, indicating that high plasmid gene expression is further limited to a small subpopulation of cells.

AAV production necessitates the successful transfection of all three plasmids, as it would otherwise lead to low titers or incomplete viral packaging^12^. However, the efficiency of transfection typically suffers from high variability and lack of scalability^51^. scRNA seq revealed differences in plasmid gene expression between individual cells originating from the same sample and showed that a large proportion of cells lacked expression of at least one plasmid. This proportion was predominantly influenced by phenotype and marginally influenced by time post-transfection, with suspension cells and later time points exhibiting a larger proportion containing all three plasmids.

A low proportion of cells containing intracellular assembled capsids was observed in the bulk sample, potentially linked to the low percentage of cells expressing high plasmid gene expression. Dash et al. had similar findings when staining for intracellular assembled capsids, with only 5–10% of transiently-transfected HEK293SF cells expressing assembled rAAV capsids, even with high transfection efficiencies and vector yields^52^. These findings, combined with the fact that only a small fraction of the suspension cells (8%) and adherent cells (5%) at 48 hpt had high expression of plasmid genes (Fig. 4), indicates that not all the cells were successfully producing rAAVs. This shows that the heterogeneity in plasmid gene expression and the low fraction of cells producing rAAVs are bottlenecks that need to be addressed to improve titers.

The clear separation of cells with different plasmid gene expressions during clustering indicated that distinct progression towards different states of plasmid gene expression could be discerned from trajectory inference. Three lineages were identified, all originating from cluster 0 which contained the largest number of cells and displayed low plasmid gene expression. One lineage, lineage β, led to the low-to-moderate plasmid gene expression in cluster 2. Another lineage, lineage α, transitioned into high plasmid gene expression. Genes that were differentially expressed along the lineages were identified and grouped into similar expression patterns, and significant GO terms associated with the groups of genes were determined.

The adherent lineage β leading to low plasmid gene expression started with terms associated with the G1 phase of the cell cycle^53–56^. Other significant GO terms include canonical Wnt signaling pathway and the regulation of the β-catenin import into the nucleus. The canonical Wnt signaling pathway is responsible for G1- to S-phase progression^57^. This behavior suggests the progression of the cells from G1 to S to G2/M phase. GO terms associated with the regulation of the immune response and response to incorrect protein folding were upregulated at the end of the lineage.

In the lineage α of adherent cells which leads to high plasmid gene expression, GO terms relating to the M and G1/S phases of the cell cycle were cyclically expressed in the lineage, and underwent two cycles of upregulation and downregulation. Positive regulation of transmembrane receptor protein serine/threonine kinase signaling pathway, which is highly associated with the M phase^58^, was upregulated in the beginning of the lineage, followed by downregulation. Lineage α displayed a high upregulation of ion transmembrane transport at the end, specifically for potassium, which has been linked to G1/S transition^59^. These findings potentially show the progression of cells from M- to G1- early on and then finally to the S-phase. Similar to the lineage β of adherent cells, GO terms related to the immune response were also upregulated at the end of the lineage.

The lineage β of suspension cells leading to low plasmid gene expression showed some cyclical upregulation and downregulation, but most groups were either upregulated at the beginning or the end of the lineage. M- and G1-phase related GO terms such as mitosis, generation of precursor metabolites and cell size regulation were cyclically expressed, and both were downregulated at the end of the lineage. The negative regulation of the cell cycle observed at the end of the lineage could potentially indicate an arrest in the M-phase.

Reminiscent of the lineage β of adherent cells, incorrect protein folding response was also observed for the suspension lineage β. The lineages leading to low plasmid gene expression were the only ones displaying incorrect protein folding, suggesting that this could be a potential reason for the low plasmid gene levels. Other omics studies also confirm the association of protein folding with rAAV production^15–18,25^. The outcome herein shows that protein folding could be optimized further to prevent limitations caused by incorrectly folded protein.

In the lineage α of suspension cells leading to high plasmid gene expression, GO terms related to the M-phase such as mitosis and chromosome segregation were upregulated at early pseudotime values for lineage α. In line with this, an upregulation of GO terms related to the G1-phase such as cell growth and metabolism was observed. The gamma aminobutyric acid signaling pathway upregulated at the end of the lineage has been shown to lead to an accumulation of cells in the S-phase^60^. These results indicate the transition of cells from M- to G1- to S-phase for the lineage α of suspension cells, a behavior which is also seen in the lineage α of adherent cells.

Generally, an earlier immune response was activated for the β lineages leading to low plasmid gene expression. The α lineages leading to high plasmid gene expression also triggered the immune response, although at a much later time point and nearing the end of the lineage. This outcome indicates that the regulation or the delay of the immune response may be a valid strategy for enhancing rAAV productivity.

The link between wild type AAV expansion *in vivo* and the cell cycle has been previously established. AAVs have been found to modulate the host cell cycle to facilitate viral genome replication, which mainly occurs in the S/G2 phase. Indeed, our data demonstrate that the cluster of cells exhibiting high plasmid expression also showed significant upregulation of the S-phase gene signature whereas the G2M-phase signature showed a less pronounced correlation, as shown in Figures S7 and S8^61^. Franzoso et al. showed that AAV2 gene expression occurs exclusively in cells in the S/G2 phases due to the cell cycle dependent expression of the Rep genes^62^. Rep 78 has been shown to activate caspase-3, which induces apoptosis during the G_1_ and early S phases of the cell cycle^63^, as well as to decrease the Cdc25A activity and accumulate pRb, which induces a complete arrest of the cells in the S phase^64,65^. Additionally, many other genes involved in rAAV production induce entry into the S phase or freeze the cells in the S phase by preventing mitosis, apoptosis such as the E1 and E4 genes^66,67^. The host cell DNA replication machinery required by DNA viruses for replication is only accessible during the S phase. To make the environment favorable for viral replication, AAVs must induce S-phase entry of infected cells, which they achieve through the virally-encoded proteins that induce S phase entry^68^.

The influence of the cell cycle on rAAV productivity was partly shown by Barnes et al., who showed that modifying the SKA2 and ITPRIP gene expressions, which lead to modulatory effects on the cell cycle increased rAAV genome titers^69^. Another study by Tworig et al. who performed bulk RNA seq during rAAV production found the upregulation of proliferation inhibitors and downregulation of proliferation enhancers among high AAV producer cell lines, and proposed modulation of the cell cycle as a strategy to improve AAV titers^70^. Additionally, a study performed by Budge on plasmid DNA delivery during transfection of both CHO and HEK293 cells highlighted that plasmid DNA dispersal and nuclear import are facilitated by cell division, especially for PEI-mediated transfection, and that cell cycle synchronization to maximize cell division enhances transfection efficiency^71^. The findings in the present study show a strong link between high plasmid gene expression and the cell cycle. Cell cycle synchronization could be used as a strategy for improving plasmid gene expression and thus rAAV production.

## Conclusion

This study reports the first application of single-cell transcriptomics on transient transfection-based rAAV production with adherent and suspension HEK293T cells. In our effort to characterize the process, we uncovered the substantial heterogeneity in HEK293 cells during production and identified subgroups of cells with high and low rAAV plasmid gene expression.

Major differences in plasmid gene expression were observed between the cells, with nearly half of the cells missing expression of genes from at least one plasmid, suggesting that successful plasmid gene expression is a significant bottleneck for the production. Additionally, we determined that a low proportion of cells produce intracellular assembled capsids. Key factors that distinguished cells with high and low plasmid gene expression were identified, with cell cycle being the primary contributor. Reducing the heterogeneity through strategies such as cell cycle synchronization could potentially improve the productivity of rAAV production based on transient transfection of HEK293 cells with producer plasmids. Further study in this direction is ongoing. This study contributes to our biological understanding of the production process as well as emphasizes the need for further omics studies.

## Supplementary Information

Below is the link to the electronic supplementary material.

Supplementary Material 1

## Data Availability

The datasets analysed during the current study are available in the Sequencing Read Archive (SRA) with the accession number http://www.ncbi.nlm.nih.gov/bioproject/1236707.

## Abbreviations

rAAV: recombinant adeno associated virus
RNA seq: RNA sequencing
scRNA seq: single-cell RNA sequencing
PEI: Polyethylenimine
GFP: Green fluorescent protein
HPT: Hours post transfection
DMSO: Dimethylsulfoxide
PBS: Phosphate-buffered saline
PBST: Phosphate-buffered saline with 0.1% Tween 20
SV40LT: Simian virus 40 large T antigen
UMI: Unique molecular identifier
ADH: Adherent
SUP: Suspension
