# Supplementary material for: Heterogeneity in an adeno-associated virus transfection-based production process limits the production efficiency
Source: Sci Rep. 2025 Nov 4;15:38459. doi: 10.1038/s41598-025-26261-0 (PMC12586574; doi:10.1038/s41598-025-26261-0)
Supplement: Supplementary file 1 — Supplementary Material 1 [file 41598_2025_26261_MOESM1_ESM.docx]

# Supplementary information

**Table S1** Number of cells and median reads per cell for each sample used in the single cell RNA sequencing experiment.

| **Samples** | **Short name** | **Number of cells** | **Median reads per cell** |
| --- | --- | --- | --- |
| Adherent 12 hpt | AD_12hpt | 3189 | 37826 |
| Adherent 48 hpt | AD_48hpt | 2648 | 38260 |
| Suspension 12 hpt | SUP_12hpt | 2307 | 36437 |
| Suspension 48 hpt | SUP_48hpt | 1809 | 41138 |

**Table S2** Normalized counts of mapped reads for all samples used in the bulk RNA sequencing experiment.

| **Samples** | **Short name** | **Normalized counts** |
| --- | --- | --- |
| Adherent 0 hpt Replicate 1 | AD_0hpt_R1 | 25878059 |
| Adherent 0 hpt Replicate 2 | AD_0hpt_R2 | 25652991 |
| Adherent 0 hpt Replicate 3 | AD_0hpt_R3 | 25911753 |
| Adherent 0 hpt Replicate 4 | AD_0hpt_R4 | 26015992 |
| Adherent 12 hpt Replicate 1 | AD_12hpt_R1 | 26165996 |
| Adherent 12 hpt Replicate 2 | AD_12hpt_R2 | 26203756 |
| Adherent 12 hpt Replicate 3 | AD_12hpt_R3 | 25873929 |
| Adherent 12 hpt Replicate 4 | AD_12hpt_R4 | 25596065 |
| Adherent 24 hpt Replicate 1 | AD_24hpt_R1 | 25213562 |
| Adherent 24 hpt Replicate 2 | AD_24hpt_R2 | 25875383 |
| Adherent 24 hpt Replicate 3 | AD_24hpt_R3 | 28064979 |
| Adherent 24 hpt Replicate 4 | AD_24hpt_R4 | 26055895 |
| Adherent 48 hpt Replicate 1 | AD_48hpt_R1 | 26311699 |
| Adherent 48 hpt Replicate 2 | AD_48hpt_R2 | 26179026 |
| Adherent 48 hpt Replicate 3 | AD_48hpt_R3 | 26351898 |
| Adherent 48 hpt Replicate 4 | AD_48hpt_R4 | 26057152 |
| Suspension 0 hpt Replicate 1 | SUP_0hpt_R1 | 27107369 |
| Suspension 0 hpt Replicate 2 | SUP_0hpt_R2 | 27230766 |
| Suspension 0 hpt Replicate 3 | SUP_0hpt_R3 | 26891270 |
| Suspension 0 hpt Replicate 4 | SUP_0hpt_R4 | 26926757 |
| Suspension 12 hpt Replicate 1 | SUP_12hpt_R1 | 27366718 |
| Suspension 12 hpt Replicate 2 | SUP_12hpt_R2 | 27101081 |
| Suspension 12 hpt Replicate 3 | SUP_12hpt_R3 | 27343421 |
| Suspension 12 hpt Replicate 4 | SUP_12hpt_R4 | 27922820 |
| Suspension 24 hpt Replicate 1 | SUP_24hpt_R1 | 27683416 |
| Suspension 24 hpt Replicate 2 | SUP_24hpt_R2 | 27613585 |
| Suspension 24 hpt Replicate 3 | SUP_24hpt_R3 | 27732712 |
| Suspension 24 hpt Replicate 4 | SUP_24hpt_R4 | 27807411 |
| Suspension 48 hpt Replicate 1 | SUP_48hpt_R1 | 27420393 |
| Suspension 48 hpt Replicate 2 | SUP_48hpt_R2 | 26957645 |
| Suspension 48 hpt Replicate 3 | SUP_48hpt_R3 | 27403067 |
| Suspension 48 hpt Replicate 4 | SUP_48hpt_R4 | 27565075 |

| 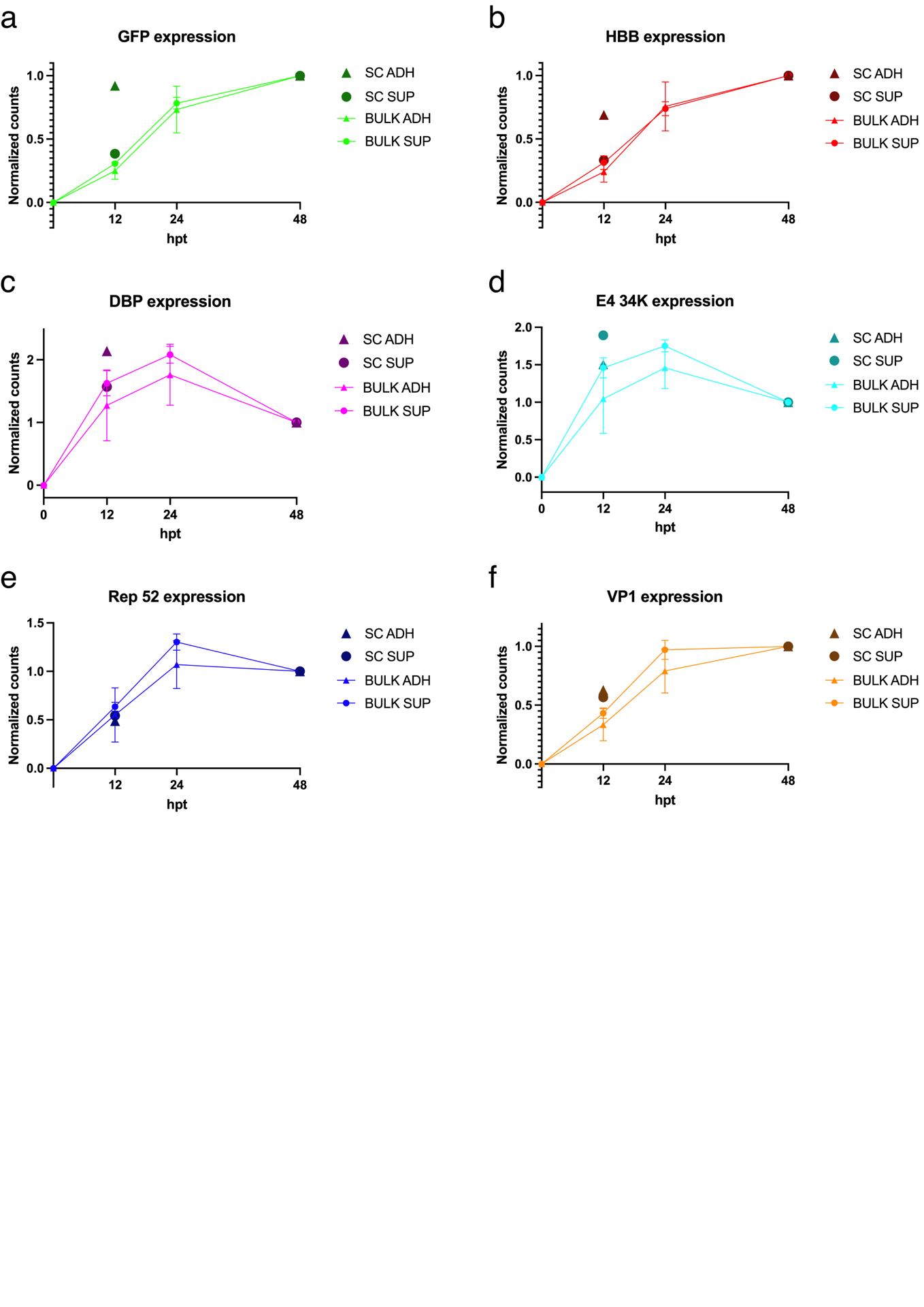 |
| --- |
| **Figure S1** Normalized counts from the bulk (BULK) RNA sequencing and single cell (SC) RNA sequencing experiments of the plasmid genes GFP (a), HBB (b), DBP (c), E4 34K (d), Rep 52 (e) and VP1 (f). |

| 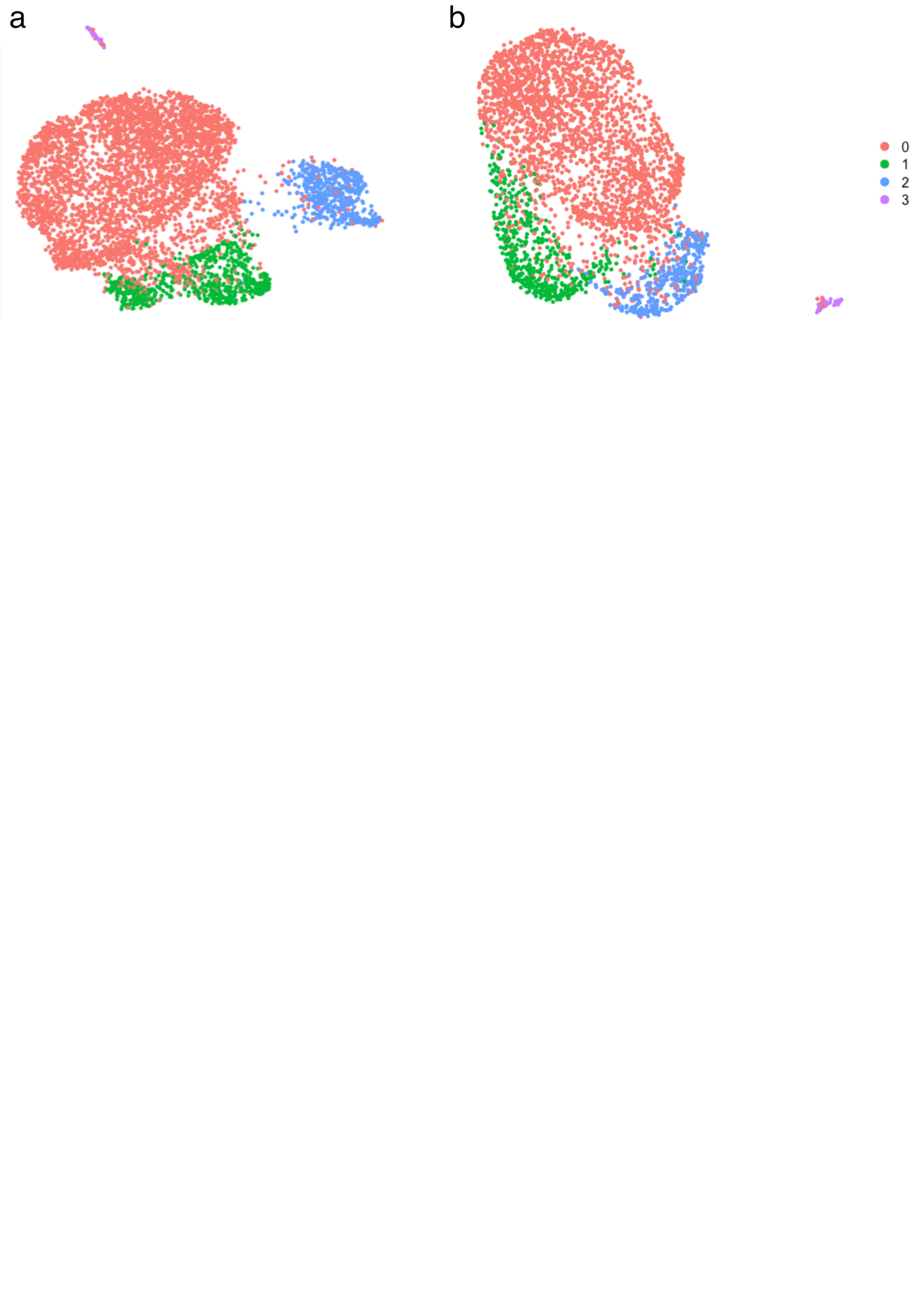 |
| --- |
| **Figure S2** UMAP plot of the adherent (a) and suspension (b) subsets. Each point corresponds to a cell. Four distinct clusters were obtained for both subsets: 0 (red), 1 (green), 2 (blue), 3 (purple). |

| 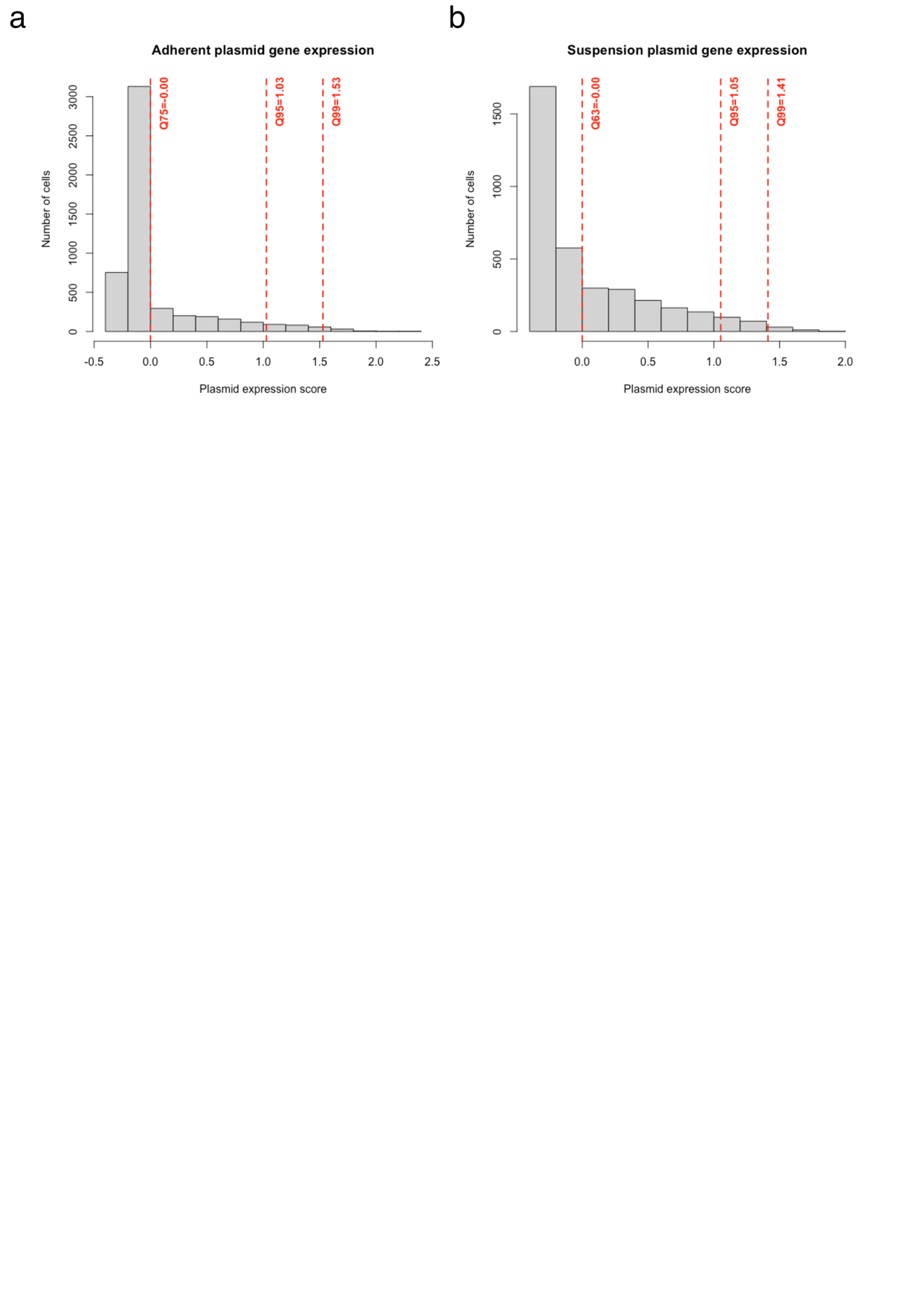 |
| --- |
| **Figure S3** Histogram of the module score for the plasmid gene expression in the adherent (a) and suspension (b) subsets. |

| 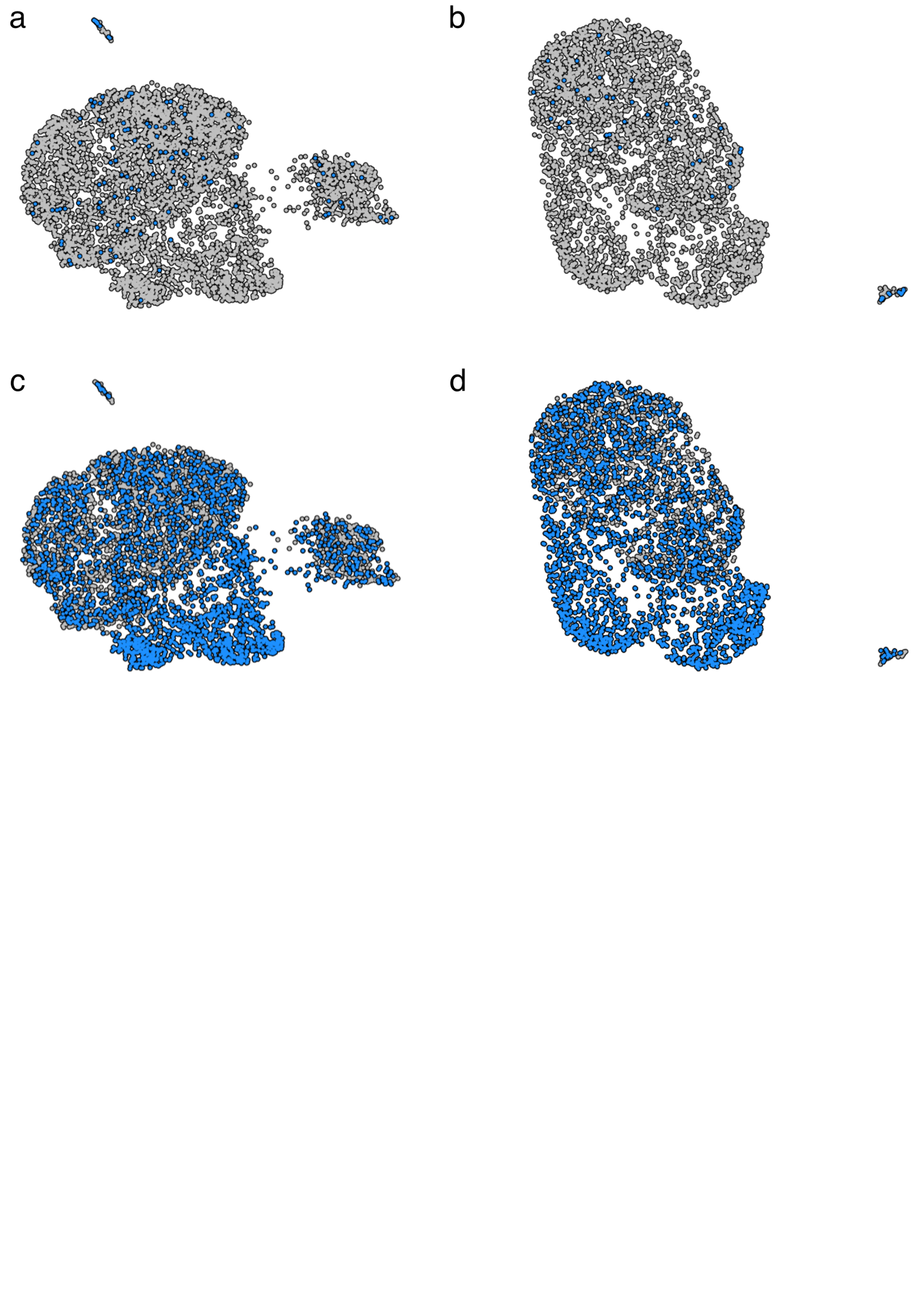 |
| --- |
| **Figure S4** UMAP plot where each point corresponds to a cell (grey or blue) for adherent (a and c) and suspension (b and d) subsets. Highlighted cells (blue) show cells with no plasmid gene expression (a and b) or cells expressing all plasmid genes (c and d) for each subset. |

| 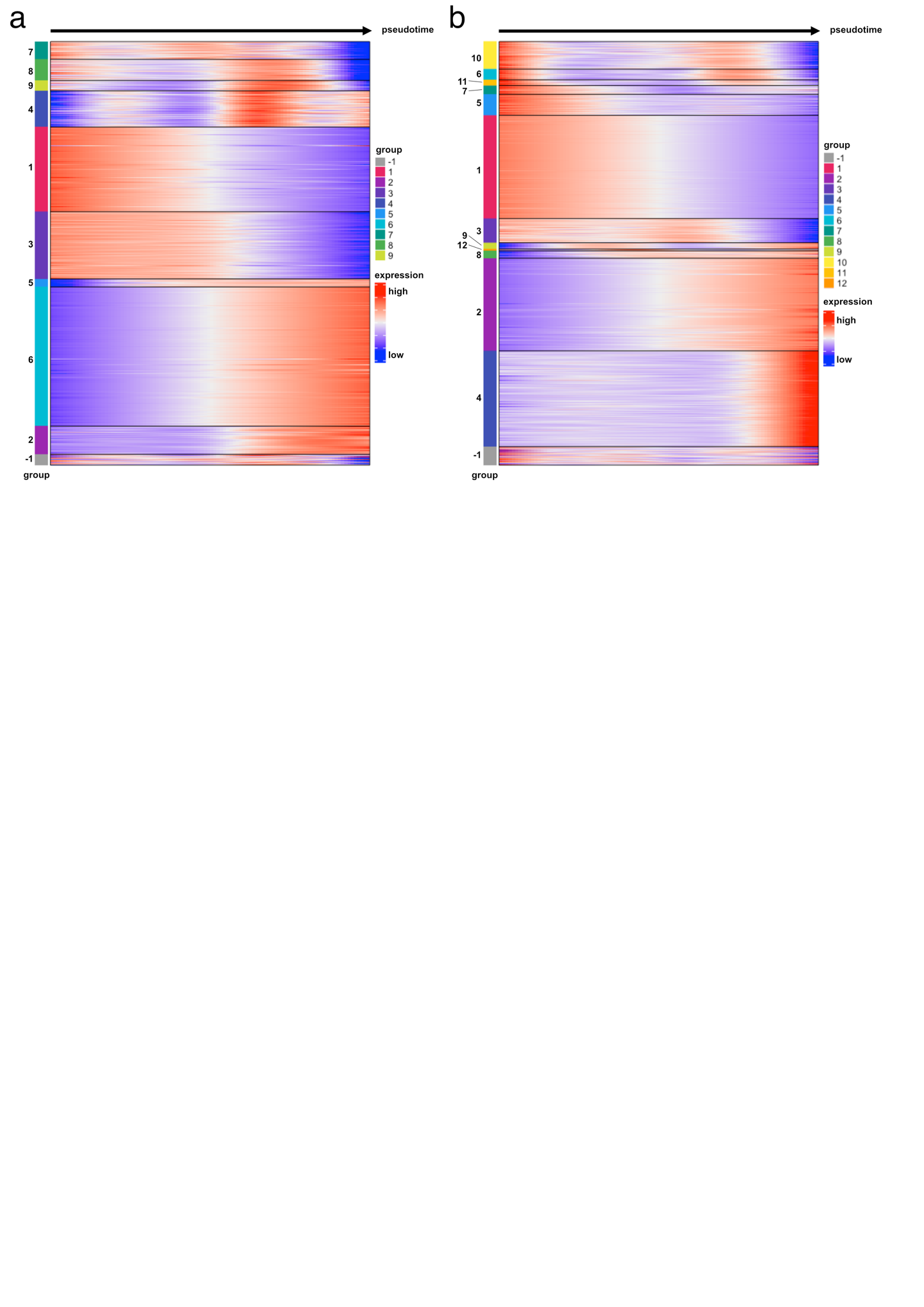 |
| --- |
| **Figure S5** Heatmap plot of the relative expression levels (log2 normalized z-score) of differentially expressed genes associated with the trajectory of adherent lineages towards low (a) or high (b) plasmid gene expression. The genes are grouped based on expression pattern over pseudotime. The group labelled -1 shows genes that were not successfully grouped. |

| 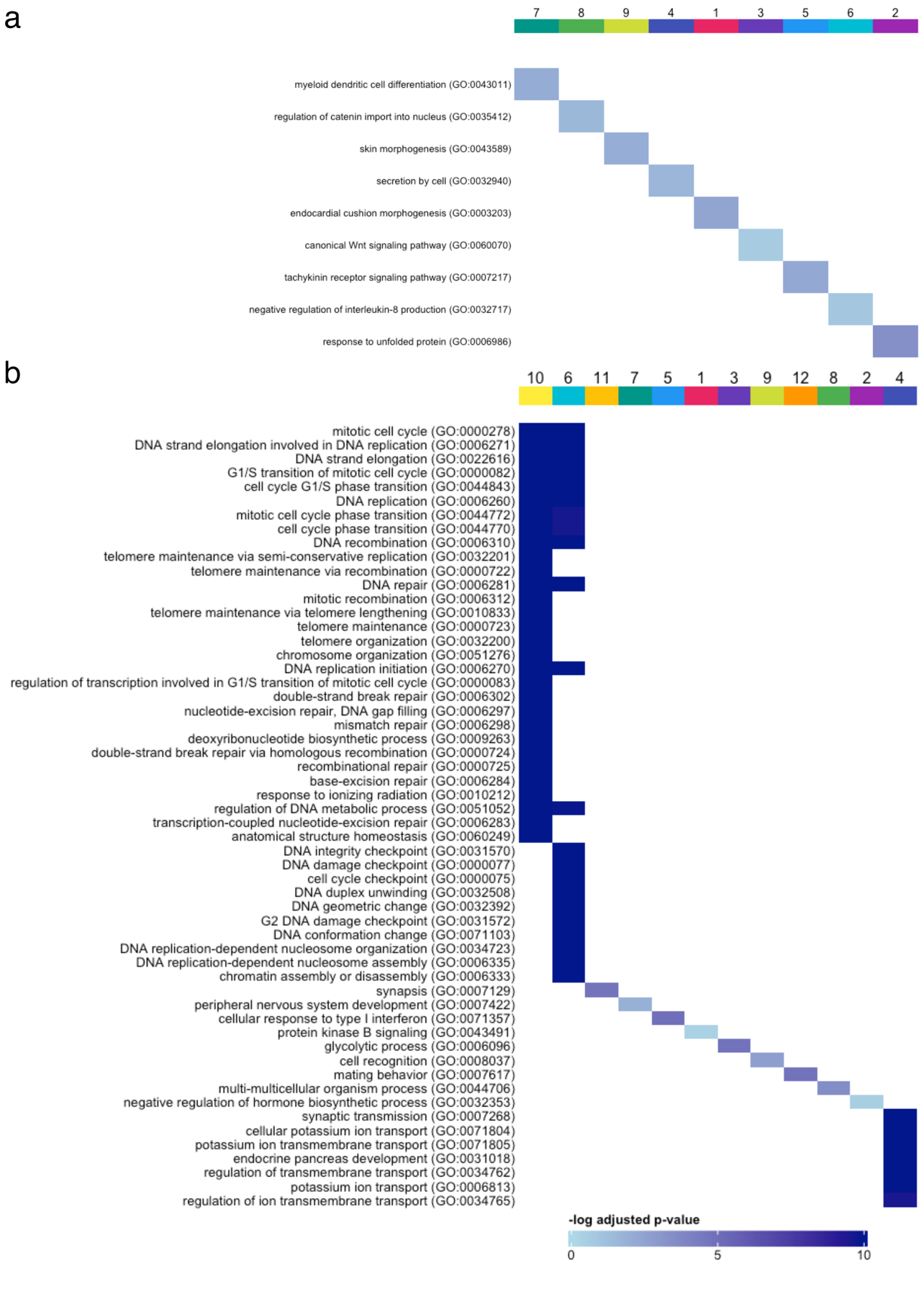 |
| --- |
| **Figure S6** Results from the overrepresentation analysis with Gene Ontology biological processes of groups of differentially expressed genes associated with the adherent lineages towards low (a) or high (b) plasmid gene expression. The genes are grouped based on expression pattern over pseudotime. |

| 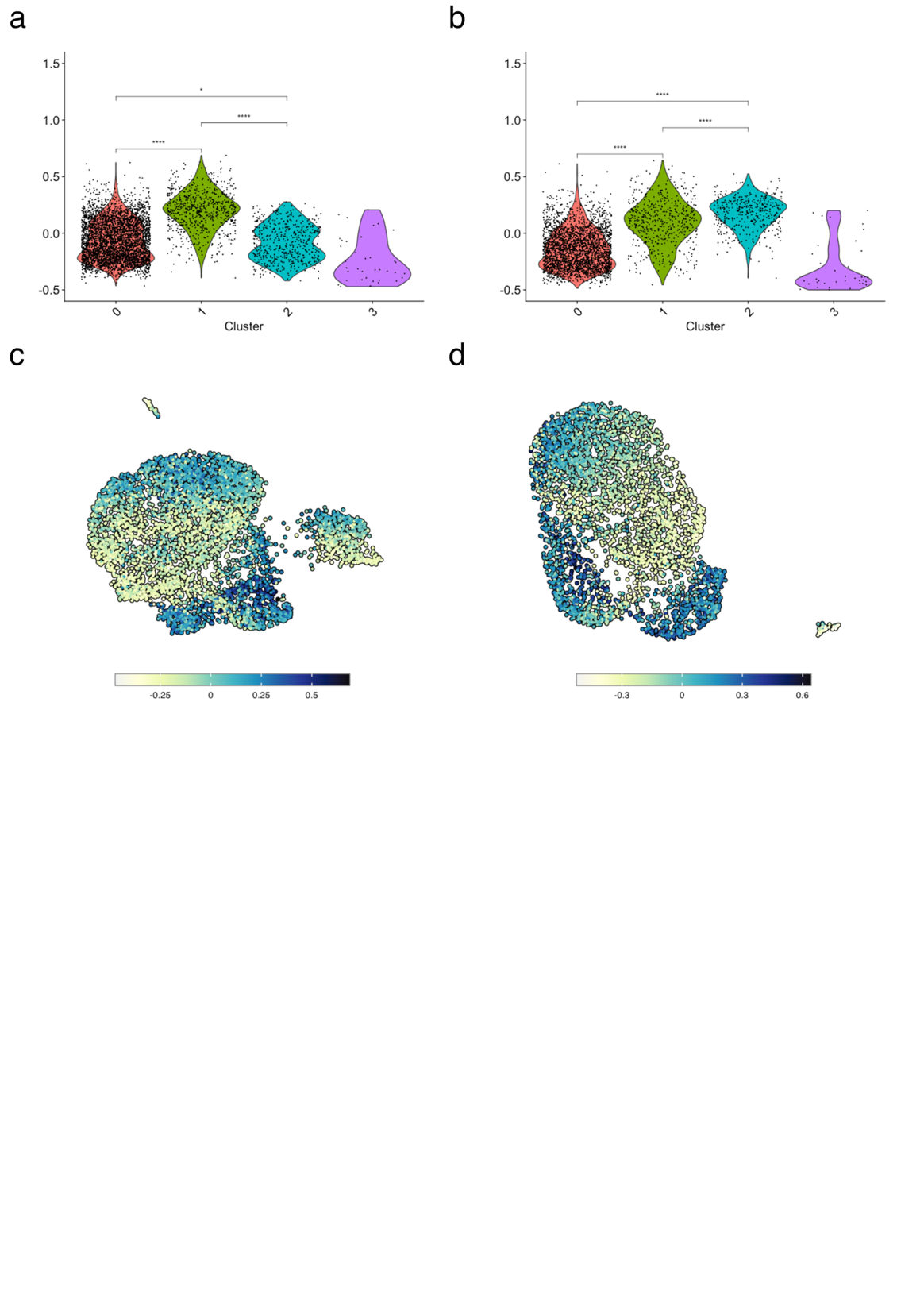 |
| --- |
| **Figure S7** Violin plot of adherent (a) and suspension (b) subsets of the single cell RNA sequencing data showing the S-phase score of each cluster. UMAP plot highlighting the plasmid gene expression score in the adherent (c) and suspension (d) subsets. |

| 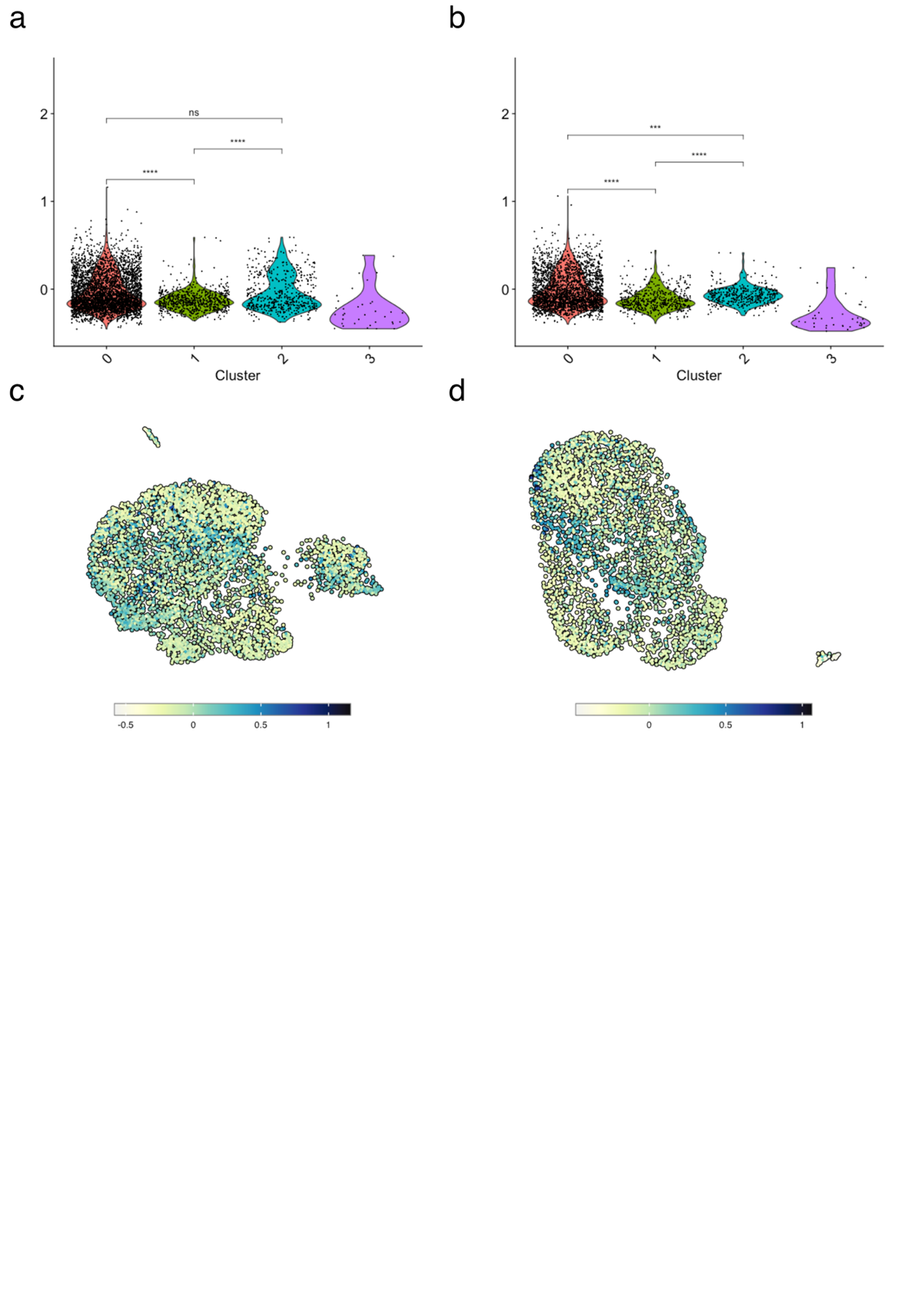 |
| --- |
| **Figure S8** Violin plot of adherent (a) and suspension (b) subsets of the single cell RNA sequencing data showing the G2M-phase score of each cluster. UMAP plot highlighting the plasmid gene expression score in the adherent (c) and suspension (d) subsets. |

**Table S3** Gene Ontology biological processes for the groups of differentially expressed genes associated with the trajectory of the adherent lineage towards low plasmid gene expression.

| **Term** | **Overlap** | **P.value** | **Adjusted.P.value** | **Odds.Ratio** | **Genes** | **cluster** |
| --- | --- | --- | --- | --- | --- | --- |
| **myeloid dendritic cell differentiation (GO:0043011)** | 2/17 | 5,20E-04 | 1,35E-01 | 69,98246 | TNFSF9;TGFBR2 | 7 |
| **myeloid dendritic cell activation (GO:0001773)** | 2/20 | 7,24E-04 | 1,35E-01 | 58,30994 | TNFSF9;TGFBR2 | 7 |
| **cerebellum development (GO:0021549)** | 2/30 | 1,64E-03 | 1,52E-01 | 37,46617 | ZBTB18;SSTR2 | 7 |
| **regulation of catenin import into nucleus (GO:0035412)** | 2/23 | 9,13E-04 | 2,30E-01 | 51,32561 | SEMA5A;DACT3 | 8 |
| **regulation of cardiac muscle tissue growth (GO:0055021)** | 2/35 | 2,12E-03 | 2,30E-01 | 32,64210 | COL14A1;FGF20 | 8 |
| **patterning of blood vessels (GO:0001569)** | 2/35 | 2,12E-03 | 2,30E-01 | 32,64210 | SEMA5A;PLXND1 | 8 |
| **skin morphogenesis (GO:0043589)** | 1/8 | 8,37E-03 | 1,42E-01 | 142,65714 | GBA | 9 |
| **negative regulation of cytosolic calcium ion concentration (GO:0051481)** | 1/8 | 8,37E-03 | 1,42E-01 | 142,65714 | OPRM1 | 9 |
| **negative regulation of cAMP-mediated signaling (GO:0043951)** | 1/8 | 8,37E-03 | 1,42E-01 | 142,65714 | OPRM1 | 9 |
| **secretion by cell (GO:0032940)** | 7/415 | 9,29E-04 | 2,14E-01 | 4,92427 | LAT2;HCK;HSPA8;SERPINE1;PDGFB;CD36;ENG | 4 |
| **defense response to Gram-positive bacterium (GO:0050830)** | 3/52 | 9,81E-04 | 2,14E-01 | 16,90136 | HCK;C5AR1;CD36 | 4 |
| **positive regulation of homeostatic process (GO:0032846)** | 4/130 | 1,45E-03 | 2,14E-01 | 8,85267 | P2RX7;INPP5K;TAC1;HSPA1A | 4 |
| **endocardial cushion morphogenesis (GO:0003203)** | 3/9 | 6,59E-05 | 9,85E-02 | 53,52973 | HEY1;SOX9;ISL1 | 1 |
| **mesenchyme morphogenesis (GO:0072132)** | 3/12 | 1,69E-04 | 1,26E-01 | 35,68108 | HEY1;SOX9;ISL1 | 1 |
| **regulation of cellular component size (GO:0032535)** | 7/165 | 9,67E-04 | 3,31E-01 | 4,81076 | MAGEL2;MYADM;MLST8;NEFL;SH3BGRL3;NEFM;SLIT2 | 1 |
| **canonical Wnt signaling pathway (GO:0060070)** | 5/84 | 6,39E-04 | 5,84E-01 | 7,86442 | JUP;FZD7;DVL2;FZD10;SOX4 | 3 |
| **heart valve morphogenesis (GO:0003179)** | 3/24 | 9,65E-04 | 5,84E-01 | 17,58208 | JUP;TWIST1;SOX4 | 3 |
| **non-canonical Wnt signaling pathway (GO:0035567)** | 3/29 | 1,69E-03 | 5,84E-01 | 14,19732 | FZD7;DVL2;FZD10 | 3 |
| **tachykinin receptor signaling pathway (GO:0007217)** | 1/8 | 5,59E-03 | 1,11E-01 | 219,54945 | TAC3 | 5 |
| **positive regulation of tyrosine phosphorylation of Stat1 protein (GO:0042511)** | 1/9 | 6,28E-03 | 1,11E-01 | 192,09615 | HPX | 5 |
| **heme transport (GO:0015886)** | 1/9 | 6,28E-03 | 1,11E-01 | 192,09615 | HPX | 5 |
| **negative regulation of interleukin-8 production (GO:0032717)** | 3/14 | 4,98E-04 | 4,64E-01 | 23,73929 | OTUD7B;SSC5D;ELANE | 6 |
| **muscle filament sliding (GO:0030049)** | 4/38 | 9,25E-04 | 4,64E-01 | 10,27382 | ACTA1;MYH3;TCAP;NEB | 6 |
| **actin-myosin filament sliding (GO:0033275)** | 4/38 | 9,25E-04 | 4,64E-01 | 10,27382 | ACTA1;MYH3;TCAP;NEB | 6 |
| **response to unfolded protein (GO:0006986)** | 5/135 | 8,98E-05 | 3,51E-02 | 12,28474 | DNAJB1;HSPA6;ASNS;CHAC1;ATF3 | 2 |
| **response to topologically incorrect protein (GO:0035966)** | 5/143 | 1,18E-04 | 3,51E-02 | 11,56791 | DNAJB1;HSPA6;ASNS;CHAC1;ATF3 | 2 |
| **protein refolding (GO:0042026)** | 2/19 | 1,82E-03 | 3,62E-01 | 36,04706 | DNAJB1;HSPA6 | 2 |

**Table S4** Gene Ontology biological processes for the groups of differentially expressed genes associated with the trajectory of the adherent lineage towards high plasmid gene expression.

| **Term** | **Overlap** | **P.value** | **Adjusted.P.value** | **Odds.Ratio** | **Genes** | **cluster** |
| --- | --- | --- | --- | --- | --- | --- |
| **mitotic cell cycle (GO:0000278)** | 29/404 | 9,08E-30 | 8,64E-27 | 32,16573 | PRIM2;FEN1;PCNA;GMNN;PKMYT1;TYMS;MYBL2;NUP43;FBXO5;RFC5;CENPU;GINS2;RRM1;RFC4;RRM2;XRCC2;GINS4;RPA1;RPA2;CDC25A;DHFR;POLA2;CCNE2;CCNE1;CDK2;CENPK;MCM4;NCAPD3;MCM2 | 10 |
| **DNA strand elongation involved in DNA replication (GO:0006271)** | 12/33 | 1,21E-21 | 5,77E-19 | 177,70536 | RFC5;PRIM2;GINS2;FEN1;POLA2;PCNA;RFC4;GINS4;RPA1;RPA2;MCM4;MCM2 | 10 |
| **DNA strand elongation (GO:0022616)** | 12/35 | 2,83E-21 | 8,99E-19 | 162,23641 | RFC5;PRIM2;GINS2;FEN1;POLA2;PCNA;RFC4;GINS4;RPA1;RPA2;MCM4;MCM2 | 10 |
| **G1/S transition of mitotic cell cycle (GO:0000082)** | 17/152 | 9,80E-21 | 1,87E-18 | 42,23641 | PRIM2;PCNA;RRM2;RPA1;RPA2;PKMYT1;TYMS;CDC25A;DHFR;POLA2;CCNE2;CCNE1;CDK2;RBBP8;MCM4;FBXO5;MCM2 | 10 |
| **cell cycle G1/S phase transition (GO:0044843)** | 17/152 | 9,80E-21 | 1,87E-18 | 42,23641 | PRIM2;PCNA;RRM2;RPA1;RPA2;PKMYT1;TYMS;CDC25A;DHFR;POLA2;CCNE2;CCNE1;CDK2;RBBP8;MCM4;FBXO5;MCM2 | 10 |
| **DNA replication (GO:0006260)** | 16/186 | 1,08E-17 | 1,68E-15 | 30,98667 | RFC5;GINS2;FEN1;DUT;RRM1;RFC4;RRM2;DSCC1;GINS4;RPA1;RPA2;CDC25A;POLA2;CDK2;MCM4;MCM2 | 10 |
| **mitotic cell cycle phase transition (GO:0044772)** | 18/277 | 1,23E-17 | 1,68E-15 | 23,56344 | PRIM2;PCNA;RRM2;RPA1;RPA2;PKMYT1;TYMS;CDC25A;DHFR;POLA2;CCNE2;CCNE1;CDK2;RBBP8;MCM4;MYBL2;FBXO5;MCM2 | 10 |
| **cell cycle phase transition (GO:0044770)** | 18/280 | 1,49E-17 | 1,78E-15 | 23,29008 | PRIM2;PCNA;RRM2;RPA1;RPA2;PKMYT1;TYMS;CDC25A;DHFR;POLA2;CCNE2;CCNE1;CDK2;RBBP8;MCM4;MYBL2;FBXO5;MCM2 | 10 |
| **DNA recombination (GO:0006310)** | 15/196 | 7,23E-16 | 7,65E-14 | 26,82230 | RFC5;PRIM2;FEN1;RFC4;PCNA;XRCC2;RPA1;RPA2;UNG;PSMC3IP;RAD51AP1;POLA2;MSH2;RBBP8;RAD54L | 10 |
| **telomere maintenance via semi-conservative replication (GO:0032201)** | 8/22 | 9,11E-15 | 8,67E-13 | 167,31092 | RFC5;PRIM2;FEN1;POLA2;PCNA;RFC4;RPA1;RPA2 | 10 |
| **telomere maintenance via recombination (GO:0000722)** | 8/26 | 4,40E-14 | 3,81E-12 | 130,10458 | RFC5;PRIM2;FEN1;POLA2;PCNA;RFC4;RPA1;RPA2 | 10 |
| **DNA repair (GO:0006281)** | 17/403 | 1,37E-13 | 1,09E-11 | 14,58444 | RFC5;FEN1;RFC4;PCNA;XRCC2;RPA1;RPA2;UNG;RAD51AP1;MSH2;UBE2T;CDK2;RBBP8;USP1;RAD54L;TOPBP1;TP73 | 10 |
| **mitotic recombination (GO:0006312)** | 8/31 | 2,19E-13 | 1,60E-11 | 101,79540 | RFC5;PRIM2;FEN1;POLA2;PCNA;RFC4;RPA1;RPA2 | 10 |
| **telomere maintenance via telomere lengthening (GO:0010833)** | 8/36 | 8,26E-13 | 5,62E-11 | 83,59664 | RFC5;PRIM2;FEN1;POLA2;PCNA;RFC4;RPA1;RPA2 | 10 |
| **telomere maintenance (GO:0000723)** | 8/62 | 8,54E-11 | 5,42E-09 | 43,28976 | RFC5;PRIM2;FEN1;POLA2;RFC4;PCNA;RPA1;RPA2 | 10 |
| **telomere organization (GO:0032200)** | 8/63 | 9,75E-11 | 5,80E-09 | 42,50053 | RFC5;PRIM2;FEN1;POLA2;RFC4;PCNA;RPA1;RPA2 | 10 |
| **chromosome organization (GO:0051276)** | 11/204 | 3,05E-10 | 1,66E-08 | 17,30100 | RFC5;PRIM2;FEN1;POLA2;RFC4;PCNA;MSH2;RPA1;RAD54L;RPA2;NCAPD3 | 10 |
| **DNA replication initiation (GO:0006270)** | 6/24 | 3,14E-10 | 1,66E-08 | 94,79048 | PRIM2;POLA2;CCNE2;CCNE1;MCM4;MCM2 | 10 |
| **regulation of transcription involved in G1/S transition of mitotic cell cycle (GO:0000083)** | 6/26 | 5,33E-10 | 2,67E-08 | 85,30286 | DHFR;PCNA;RRM2;CCNE1;FBXO5;TYMS | 10 |
| **double-strand break repair (GO:0006302)** | 8/114 | 1,19E-08 | 5,65E-07 | 21,99556 | RAD51AP1;FEN1;MSH2;XRCC2;RBBP8;RPA1;RAD54L;RPA2 | 10 |
| **nucleotide-excision repair, DNA gap filling (GO:0006297)** | 5/22 | 1,74E-08 | 7,87E-07 | 82,46479 | RFC5;PCNA;RFC4;RPA1;RPA2 | 10 |
| **mismatch repair (GO:0006298)** | 5/29 | 7,67E-08 | 3,32E-06 | 58,39202 | PCNA;MSH2;RPA1;RPA2;TP73 | 10 |
| **deoxyribonucleotide biosynthetic process (GO:0009263)** | 4/12 | 9,31E-08 | 3,85E-06 | 138,30556 | DUT;RRM1;RRM2;TYMS | 10 |
| **double-strand break repair via homologous recombination (GO:0000724)** | 6/61 | 1,16E-07 | 4,59E-06 | 30,96468 | RAD51AP1;XRCC2;RBBP8;RPA1;RAD54L;RPA2 | 10 |
| **recombinational repair (GO:0000725)** | 6/62 | 1,28E-07 | 4,87E-06 | 30,41020 | RAD51AP1;XRCC2;RBBP8;RPA1;RAD54L;RPA2 | 10 |
| **base-excision repair (GO:0006284)** | 5/42 | 5,28E-07 | 1,89E-05 | 37,85116 | FEN1;PCNA;RPA1;RPA2;UNG | 10 |
| **response to ionizing radiation (GO:0010212)** | 7/127 | 5,36E-07 | 1,89E-05 | 16,74251 | RAD51AP1;RRM1;MSH2;XRCC2;RAD54L;TOPBP1;TP73 | 10 |
| **regulation of DNA metabolic process (GO:0051052)** | 9/265 | 7,22E-07 | 2,45E-05 | 10,32020 | RAD51AP1;PCNA;MSH2;DSCC1;GMNN;CDK2;USP1;RPA2;DEK | 10 |
| **transcription-coupled nucleotide-excision repair (GO:0006283)** | 5/47 | 9,39E-07 | 3,08E-05 | 33,33669 | RFC5;PCNA;RFC4;RPA1;RPA2 | 10 |
| **anatomical structure homeostasis (GO:0060249)** | 8/208 | 1,23E-06 | 3,90E-05 | 11,60235 | RFC5;PRIM2;FEN1;POLA2;RFC4;PCNA;RPA1;RPA2 | 10 |
| **DNA replication (GO:0006260)** | 11/186 | 1,56E-15 | 7,09E-13 | 65,48722 | POLD3;BLM;CHAF1A;NASP;MCM3;MCM5;BRCA1;CLSPN;CDC6;MCM6;DTL | 6 |
| **DNA strand elongation involved in DNA replication (GO:0006271)** | 6/33 | 7,20E-12 | 1,59E-09 | 184,65741 | POLD3;GINS1;PRIM1;MCM3;MCM5;MCM6 | 6 |
| **DNA strand elongation (GO:0022616)** | 6/35 | 1,05E-11 | 1,59E-09 | 171,90517 | POLD3;GINS1;PRIM1;MCM3;MCM5;MCM6 | 6 |
| **DNA integrity checkpoint (GO:0031570)** | 7/86 | 3,98E-11 | 4,51E-09 | 76,63016 | BLM;BRIP1;E2F1;BRCA1;CLSPN;CDC6;DTL | 6 |
| **mitotic cell cycle (GO:0000278)** | 10/404 | 2,12E-10 | 1,92E-08 | 24,84264 | POLD3;GINS1;DSN1;PRIM1;E2F1;MCM3;MCM5;CDC6;MCM6;E2F8 | 6 |
| **DNA damage checkpoint (GO:0000077)** | 6/77 | 1,47E-09 | 1,11E-07 | 70,06690 | BLM;BRIP1;E2F1;BRCA1;CLSPN;DTL | 6 |
| **cell cycle checkpoint (GO:0000075)** | 7/156 | 2,69E-09 | 1,74E-07 | 40,48643 | BLM;BRIP1;E2F1;BRCA1;CLSPN;CDC6;DTL | 6 |
| **DNA repair (GO:0006281)** | 9/403 | 4,93E-09 | 2,79E-07 | 21,29369 | POLD3;MSH6;BLM;BRIP1;CHAF1A;EXO1;BRCA1;CLSPN;DTL | 6 |
| **DNA duplex unwinding (GO:0032508)** | 5/55 | 1,77E-08 | 8,88E-07 | 79,68000 | BLM;BRIP1;MCM3;MCM5;MCM6 | 6 |
| **DNA geometric change (GO:0032392)** | 5/57 | 2,12E-08 | 9,60E-07 | 76,60769 | BLM;BRIP1;MCM3;MCM5;MCM6 | 6 |
| **DNA replication initiation (GO:0006270)** | 4/24 | 4,28E-08 | 1,76E-06 | 153,46154 | PRIM1;MCM3;MCM5;MCM6 | 6 |
| **cell cycle G1/S phase transition (GO:0044843)** | 6/152 | 8,91E-08 | 3,11E-06 | 33,94521 | PRIM1;E2F1;MCM3;MCM5;CDC6;MCM6 | 6 |
| **G1/S transition of mitotic cell cycle (GO:0000082)** | 6/152 | 8,91E-08 | 3,11E-06 | 33,94521 | PRIM1;E2F1;MCM3;MCM5;CDC6;MCM6 | 6 |
| **G2 DNA damage checkpoint (GO:0031572)** | 4/34 | 1,85E-07 | 5,98E-06 | 102,25641 | BLM;BRCA1;CLSPN;DTL | 6 |
| **DNA conformation change (GO:0071103)** | 5/99 | 3,47E-07 | 9,67E-06 | 42,28936 | BLM;BRIP1;MCM3;MCM5;MCM6 | 6 |
| **DNA replication-dependent nucleosome organization (GO:0034723)** | 3/10 | 3,63E-07 | 9,67E-06 | 316,87302 | CHAF1A;NASP;ASF1B | 6 |
| **DNA replication-dependent nucleosome assembly (GO:0006335)** | 3/10 | 3,63E-07 | 9,67E-06 | 316,87302 | CHAF1A;NASP;ASF1B | 6 |
| **DNA recombination (GO:0006310)** | 6/196 | 4,00E-07 | 1,01E-05 | 26,02632 | POLD3;MSH6;BLM;EXO1;PRIM1;BRCA1 | 6 |
| **chromatin assembly or disassembly (GO:0006333)** | 4/58 | 1,65E-06 | 3,93E-05 | 56,74074 | HELLS;CHAF1A;NASP;ASF1B | 6 |
| **regulation of DNA metabolic process (GO:0051052)** | 6/265 | 2,32E-06 | 5,27E-05 | 19,02606 | MSH6;BLM;BRCA1;CDC6;E2F7;E2F8 | 6 |
| **mitotic cell cycle phase transition (GO:0044772)** | 6/277 | 3,00E-06 | 6,48E-05 | 18,17251 | PRIM1;E2F1;MCM3;MCM5;CDC6;MCM6 | 6 |
| **cell cycle phase transition (GO:0044770)** | 6/280 | 3,19E-06 | 6,58E-05 | 17,97080 | PRIM1;E2F1;MCM3;MCM5;CDC6;MCM6 | 6 |
| **synapsis (GO:0007129)** | 2/12 | 3,94E-05 | 9,50E-03 | 285,34286 | FANCD2;DMC1 | 11 |
| **chromosome organization involved in meiosis (GO:0070192)** | 2/36 | 3,72E-04 | 4,48E-02 | 83,82353 | FANCD2;DMC1 | 11 |
| **ovulation cycle process (GO:0022602)** | 2/78 | 1,74E-03 | 1,01E-01 | 37,42105 | ADAMTS1;DMC1 | 11 |
| **peripheral nervous system development (GO:0007422)** | 2/28 | 5,12E-04 | 1,59E-01 | 69,75524 | NRG1;GFRA3 | 7 |
| **positive regulation of transmembrane receptor protein serine/threonine kinase signaling pathway (GO:0090100)** | 2/73 | 3,44E-03 | 1,59E-01 | 25,48656 | TGFB1I1;GDF6 | 7 |
| **wound healing (GO:0042060)** | 2/75 | 3,63E-03 | 1,59E-01 | 24,78580 | NRG1;PLAT | 7 |
| **cellular response to type I interferon (GO:0071357)** | 4/65 | 3,06E-05 | 7,24E-03 | 25,56606 | IFITM2;OAS2;IFIT1;IFIT3 | 5 |
| **type I interferon signaling pathway (GO:0060337)** | 4/65 | 3,06E-05 | 7,24E-03 | 25,56606 | IFITM2;OAS2;IFIT1;IFIT3 | 5 |
| **response to type I interferon (GO:0034340)** | 4/66 | 3,25E-05 | 7,24E-03 | 25,15244 | IFITM2;OAS2;IFIT1;IFIT3 | 5 |
| **protein kinase B signaling (GO:0043491)** | 3/31 | 4,02E-03 | 7,56E-01 | 10,32962 | TMEM100;RPS6KB2;NKX3-1 | 1 |
| **anterior/posterior pattern specification (GO:0009952)** | 6/150 | 4,88E-03 | 7,56E-01 | 4,05281 | HES7;MSX2;HOXA7;BARX1;HOXC11;NKX3-1 | 1 |
| **negative regulation of epidermal cell differentiation (GO:0045605)** | 2/11 | 5,57E-03 | 7,56E-01 | 21,34088 | MSX2;HOXA7 | 1 |
| **glycolytic process (GO:0006096)** | 4/44 | 8,49E-06 | 8,79E-03 | 36,18364 | LDHA;TPI1;PGK1;GAPDH | 3 |
| **pyruvate metabolic process (GO:0006090)** | 4/71 | 5,73E-05 | 2,16E-02 | 21,57286 | LDHA;TPI1;PGK1;GAPDH | 3 |
| **cellular component disassembly (GO:0022411)** | 7/350 | 7,39E-05 | 2,16E-02 | 7,69152 | CASP7;RPL12;HMGB2;CDK1;RPL38;KIF2C;RPS2 | 3 |
| **cell recognition (GO:0008037)** | 2/109 | 1,57E-03 | 7,37E-02 | 41,29180 | OVGP1;CD36 | 9 |
| **negative regulation of multi-organism process (GO:0043901)** | 2/133 | 2,32E-03 | 7,37E-02 | 33,68617 | OVGP1;CD36 | 9 |
| **positive regulation of macrophage cytokine production (GO:0060907)** | 1/8 | 4,39E-03 | 7,37E-02 | 285,45714 | CD36 | 9 |
| **mating behavior (GO:0007617)** | 1/10 | 1,50E-03 | 9,00E-03 | 1110,44444 | NHLH2 | 12 |
| **ovulation cycle (GO:0042698)** | 1/10 | 1,50E-03 | 9,00E-03 | 1110,44444 | NHLH2 | 12 |
| **multi-organism reproductive behavior (GO:0044705)** | 1/30 | 4,49E-03 | 1,44E-02 | 344,27586 | NHLH2 | 12 |
| **multi-multicellular organism process (GO:0044706)** | 3/121 | 1,38E-04 | 3,23E-02 | 36,07446 | STS;DRD1;TAC1 | 8 |
| **multi-organism reproductive process (GO:0044703)** | 3/140 | 2,12E-04 | 3,23E-02 | 31,04171 | STS;DRD1;TAC1 | 8 |
| **neuron-neuron synaptic transmission (GO:0007270)** | 2/58 | 1,09E-03 | 7,16E-02 | 47,44524 | DRD1;CDH8 | 8 |
| **negative regulation of hormone biosynthetic process (GO:0032353)** | 2/8 | 2,27E-03 | 6,38E-01 | 36,28205 | PDE8B;BMP5 | 2 |
| **cholesterol homeostasis (GO:0042632)** | 4/61 | 2,40E-03 | 6,38E-01 | 7,70331 | CAV3;MALL;ANGPTL3;APOC3 | 2 |
| **sterol homeostasis (GO:0055092)** | 4/62 | 2,55E-03 | 6,38E-01 | 7,57011 | CAV3;MALL;ANGPTL3;APOC3 | 2 |
| **synaptic transmission (GO:0007268)** | 26/434 | 1,48E-10 | 3,42E-07 | 5,17515 | KCNC2;STXBP1;NRXN1;KCNA2;GRM1;GNG2;APOE;BSN;GRIA3;GRIA4;CHRNB2;KCNJ6;KCNH6;KCNJ11;ABCC8;KCNB2;GAD2;KCNK16;ABCC9;KCNK17;GRIN3A;SST;KCNMA1;STX1A;KCNK3;CBLN1 | 4 |
| **cellular potassium ion transport (GO:0071804)** | 13/129 | 1,66E-08 | 1,28E-05 | 8,76013 | KCNJ6;KCNH6;KCNJ11;KCNC2;ABCC8;KCNB2;HPN;KCNA2;KCNK16;ABCC9;KCNK17;KCNMA1;KCNK3 | 4 |
| **potassium ion transmembrane transport (GO:0071805)** | 13/129 | 1,66E-08 | 1,28E-05 | 8,76013 | KCNJ6;KCNH6;KCNJ11;KCNC2;ABCC8;KCNB2;HPN;KCNA2;KCNK16;ABCC9;KCNK17;KCNMA1;KCNK3 | 4 |
| **endocrine pancreas development (GO:0031018)** | 8/39 | 3,58E-08 | 2,07E-05 | 19,86391 | NEUROD1;ONECUT2;PDE3B;IAPP;NKX6-1;FOXO1;INS;NKX2-2 | 4 |
| **regulation of transmembrane transport (GO:0034762)** | 19/327 | 7,86E-08 | 3,64E-05 | 4,89176 | KCNJ6;KCNH6;KCNJ11;KCNC2;KCNB2;NRXN1;KCNA2;STC1;KCNK16;GCG;KCNK17;AGT;INS;TMEM37;DPP6;ASPH;KCNMA1;SCN3B;KCNK3 | 4 |
| **potassium ion transport (GO:0006813)** | 13/151 | 1,09E-07 | 4,21E-05 | 7,35533 | KCNJ6;KCNH6;KCNJ11;KCNC2;ABCC8;KCNB2;HPN;KCNA2;KCNK16;ABCC9;KCNK17;KCNMA1;KCNK3 | 4 |
| **regulation of ion transmembrane transport (GO:0034765)** | 18/314 | 2,13E-07 | 7,05E-05 | 4,80554 | KCNJ6;KCNH6;KCNJ11;KCNC2;KCNB2;NRXN1;KCNA2;STC1;KCNK16;GCG;KCNK17;AGT;TMEM37;DPP6;ASPH;KCNMA1;SCN3B;KCNK3 | 4 |

**Table S5** Gene Ontology biological processes for the groups of differentially expressed genes associated with the trajectory of the suspension lineage towards low plasmid gene expression.

| **Term** | **Overlap** | **P.value** | **Adjusted.P.value** | **Odds.Ratio** | **Genes** | **cluster** |
| --- | --- | --- | --- | --- | --- | --- |
| **neurofilament cytoskeleton organization (GO:0060052)** | 2/10 | 6,29E-06 | 7,36E-04 | 832,66667 | NEFL;NEFM | 14 |
| **intermediate filament organization (GO:0045109)** | 2/17 | 1,90E-05 | 1,11E-03 | 443,93333 | NEFL;NEFM | 14 |
| **intermediate filament cytoskeleton organization (GO:0045104)** | 2/37 | 9,26E-05 | 2,54E-03 | 190,06667 | NEFL;NEFM | 14 |
| **positive regulation of cell size (GO:0045793)** | 1/9 | 7,18E-03 | 1,31E-01 | 166,46667 | AMOT | 7 |
| **negative regulation of vascular permeability (GO:0043116)** | 1/11 | 8,77E-03 | 1,31E-01 | 133,16000 | AMOT | 7 |
| **cell migration involved in gastrulation (GO:0042074)** | 1/14 | 1,11E-02 | 1,31E-01 | 102,41538 | AMOT | 7 |
| **respiratory electron transport chain (GO:0022904)** | 20/112 | 2,05E-17 | 2,85E-14 | 17,79710 | COX8A;NDUFA8;COX7B;NDUFA3;NDUFB3;NDUFA2;NDUFB2;NDUFA1;NDUFB1;NDUFC1;UQCR11;COQ9;UQCR10;COX6C;COX7C;COX6B1;SCO2;UQCRQ;NDUFS6;NDUFS5 | 5 |
| **electron transport chain (GO:0022900)** | 20/115 | 3,53E-17 | 2,85E-14 | 17,23246 | COX8A;NDUFA8;COX7B;NDUFA3;NDUFB3;NDUFA2;NDUFB2;NDUFA1;NDUFB1;NDUFC1;UQCR11;COQ9;UQCR10;COX6C;COX7C;COX6B1;SCO2;UQCRQ;NDUFS6;NDUFS5 | 5 |
| **generation of precursor metabolites and energy (GO:0006091)** | 28/375 | 9,41E-14 | 5,07E-11 | 6,74506 | COX7B;COX17;NDUFB3;NDUFB2;NDUFB1;UQCR11;UQCR10;COX7C;GYS1;BLOC1S1;AKT2;SLC37A4;COX8A;NDUFA8;TPI1;NDUFA3;NDUFA2;NDUFA1;CHCHD10;NDUFC1;COQ9;COX6C;COX6B1;SCO2;UQCRQ;NDUFS6;NDUFS5;ALDOC | 5 |
| **mitochondrial electron transport, NADH to ubiquinone (GO:0006120)** | 11/45 | 9,96E-12 | 4,02E-09 | 25,60430 | NDUFA8;NDUFS6;NDUFA3;NDUFS5;NDUFB3;NDUFA2;NDUFB2;NDUFA1;NDUFB1;NDUFC1;COQ9 | 5 |
| **cofactor metabolic process (GO:0051186)** | 6/255 | 4,51E-04 | 1,72E-01 | 6,67707 | GCDH;TP53I3;UROS;RSAD1;MTHFR;NUDT12 | 6 |
| **aspartate family amino acid metabolic process (GO:0009066)** | 3/42 | 5,65E-04 | 1,72E-01 | 20,66944 | GCDH;ADI1;MTHFR | 6 |
| **methionine biosynthetic process (GO:0009086)** | 2/16 | 1,70E-03 | 1,98E-01 | 37,92190 | ADI1;MTHFR | 6 |
| **spindle checkpoint (GO:0031577)** | 3/41 | 2,87E-06 | 8,87E-04 | 143,16746 | CCNB1;PLK1;BIRC5 | 8 |
| **mitotic cell cycle (GO:0000278)** | 5/404 | 5,65E-06 | 8,87E-04 | 27,27235 | CCNB1;PTTG1;PLK1;BIRC5;KIF20A | 8 |
| **cytokinesis (GO:0000910)** | 3/79 | 2,09E-05 | 1,57E-03 | 71,44737 | PLK1;BIRC5;KIF20A | 8 |
| **phosphatidylinositol acyl-chain remodeling (GO:0036149)** | 3/16 | 1,29E-03 | 4,61E-01 | 16,60022 | PLA2G2D;PLA2G1B;PLA2G10 | 1 |
| **phosphatidylglycerol acyl-chain remodeling (GO:0036148)** | 3/17 | 1,55E-03 | 4,61E-01 | 15,41371 | PLA2G2D;PLA2G1B;PLA2G10 | 1 |
| **phosphatidylserine acyl-chain remodeling (GO:0036150)** | 3/17 | 1,55E-03 | 4,61E-01 | 15,41371 | PLA2G2D;PLA2G1B;PLA2G10 | 1 |
| **regulation of low-density lipoprotein particle receptor biosynthetic process (GO:0045714)** | 1/8 | 2,34E-02 | 5,29E-01 | 49,09852 | FGF21 | 3 |
| **response to gravity (GO:0009629)** | 1/9 | 2,62E-02 | 5,29E-01 | 42,95905 | NOX3 | 3 |
| **branching involved in labyrinthine layer morphogenesis (GO:0060670)** | 1/9 | 2,62E-02 | 5,29E-01 | 42,95905 | GCM1 | 3 |
| **respiratory burst (GO:0045730)** | 4/16 | 3,10E-06 | 3,43E-03 | 51,70833 | HCK;NCF1;NCF2;PIK3CG | 2 |
| **phagosome maturation (GO:0090382)** | 4/39 | 1,25E-04 | 6,90E-02 | 17,70804 | NCF1;NCF2;NOS1;ATP6V1G3 | 2 |
| **regulation of muscle contraction (GO:0006937)** | 5/114 | 9,59E-04 | 2,79E-01 | 7,13682 | MYOCD;NCF1;TACR2;NOS1;PIK3CG | 2 |
| **cilium or flagellum-dependent cell motility (GO:0001539)** | 2/11 | 1,00E-03 | 4,40E-01 | 52,03660 | DNAH3;DRC1 | 12 |
| **regulation of T cell receptor signaling pathway (GO:0050856)** | 2/25 | 5,26E-03 | 4,40E-01 | 20,34783 | LCK;PTPN22 | 12 |
| **regulation of antigen receptor-mediated signaling pathway (GO:0050854)** | 2/35 | 1,01E-02 | 4,40E-01 | 14,17469 | LCK;PTPN22 | 12 |
| **B cell receptor signaling pathway (GO:0050853)** | 3/33 | 7,23E-04 | 4,14E-01 | 19,09904 | LAT2;PIK3CD;NFATC2 | 13 |
| **mast cell activation involved in immune response (GO:0002279)** | 2/11 | 1,51E-03 | 4,14E-01 | 42,08254 | LAT2;PIK3CD | 13 |
| **mast cell degranulation (GO:0043303)** | 2/25 | 1,51E-03 | 4,14E-01 | 42,08254 | LAT2;PIK3CD | 13 |
| **skeletal muscle cell differentiation (GO:0035914)** | 3/47 | 1,16E-03 | 4,34E-01 | 15,93690 | MAFF;ANKRD1;PAX5 | 4 |
| **3'-phosphoadenosine 5'-phosphosulfate metabolic process (GO:0050427)** | 2/17 | 2,49E-03 | 4,34E-01 | 30,84806 | SULT2B1;SULT2A1 | 4 |
| **purine ribonucleoside bisphosphate metabolic process (GO:0034035)** | 2/17 | 2,49E-03 | 4,34E-01 | 30,84806 | SULT2B1;SULT2A1 | 4 |
| **response to alcohol (GO:0097305)** | 6/274 | 1,49E-03 | 3,52E-01 | 5,23507 | FOSL1;NPPC;STC2;FOSB;CRYAB;AQP1 | 11 |
| **cGMP biosynthetic process (GO:0006182)** | 2/16 | 2,31E-03 | 3,52E-01 | 32,29870 | NPPC;AQP1 | 11 |
| **negative regulation of cysteine-type endopeptidase activity involved in apoptotic process (GO:0043154)** | 3/69 | 3,73E-03 | 3,52E-01 | 10,36782 | DNAJB6;CRYAB;AQP1 | 11 |
| **response to unfolded protein (GO:0006986)** | 11/135 | 1,03E-10 | 1,19E-07 | 19,06583 | PPP1R15A;DNAJB1;HSP90AA1;HSPH1;HSPA4L;HSPA6;SERPINH1;CHAC1;ATF3;HERPUD1;HSPA1A | 15 |
| **response to topologically incorrect protein (GO:0035966)** | 11/143 | 1,92E-10 | 1,19E-07 | 17,90308 | PPP1R15A;DNAJB1;HSP90AA1;HSPH1;HSPA4L;HSPA6;SERPINH1;CHAC1;ATF3;HERPUD1;HSPA1A | 15 |
| **response to unfolded protein (GO:0006986)** | 11/135 | 1,03E-10 | 1,19E-07 | 19,06583 | PPP1R15A;DNAJB1;HSP90AA1;HSPH1;HSPA4L;HSPA6;SERPINH1;CHAC1;ATF3;HERPUD1;HSPA1A | 15 |
| **epithelial cell morphogenesis (GO:0003382)** | 2/38 | 5,89E-04 | 9,18E-02 | 65,17974 | GDNF;ID1 | 10 |
| **cell morphogenesis (GO:0000902)** | 3/204 | 8,98E-04 | 9,18E-02 | 18,45149 | GDNF;ID1;KLF2 | 10 |
| **regulation of monooxygenase activity (GO:0032768)** | 2/49 | 9,79E-04 | 9,18E-02 | 49,89737 | CAV3;GDNF | 10 |
| **response to mechanical stimulus (GO:0009612)** | 6/176 | 1,31E-05 | 1,46E-02 | 13,16604 | CASP8AP2;EGR1;GADD45A;TXNIP;NGF;PTGS2 | 16 |
| **negative regulation of cell cycle (GO:0045786)** | 8/430 | 3,63E-05 | 1,59E-02 | 7,25546 | BTG3;LATS2;GADD45A;NGF;PTGS2;E2F7;E2F8;TP73 | 16 |
| **cellular response to mechanical stimulus (GO:0071260)** | 4/67 | 4,56E-05 | 1,59E-02 | 22,94719 | CASP8AP2;EGR1;GADD45A;PTGS2 | 16 |
| **negative regulation of protein modification process (GO:0031400)** | 5/407 | 5,86E-06 | 2,58E-03 | 27,06468 | DUSP1;DDIT4;UBC;HSPB1;DUSP8 | 9 |
| **negative regulation of protein phosphorylation (GO:0001933)** | 4/260 | 2,52E-05 | 5,22E-03 | 30,82813 | DUSP1;DDIT4;HSPB1;DUSP8 | 9 |
| **negative regulation of transferase activity (GO:0051348)** | 4/284 | 3,56E-05 | 5,22E-03 | 28,15143 | DUSP1;UBC;HSPB1;DUSP8 | 9 |

**Table S6** Gene Ontology biological processes for the groups of differentially expressed genes associated with the trajectory of the suspension lineage towards high plasmid gene expression.

| **Term** | **Overlap** | **P.value** | **Adjusted.P.value** | **Odds.Ratio** | **Genes** | **Cluster** |
| --- | --- | --- | --- | --- | --- | --- |
| **segmentation (GO:0035282)** | 3/44 | 3,60E-04 | 1,07E-01 | 24,26341 | MAFB;WNT5A;PAX1 | 2 |
| **stem cell proliferation (GO:0072089)** | 3/48 | 4,66E-04 | 1,07E-01 | 22,10222 | BMP2;STAT1;WNT5A | 2 |
| **epithelial to mesenchymal transition (GO:0001837)** | 3/50 | 5,25E-04 | 1,07E-01 | 21,15957 | BMP2;WNT5A;SNAI2 | 2 |
| **endosomal transport (GO:0016197)** | 7/187 | 6,01E-04 | 3,79E-01 | 5,23855 | MAGEL2;TRIM27;AP5S1;CHMP7;BET1L;MTM1;RHOB | 3 |
| **cellular response to nutrient (GO:0031670)** | 3/22 | 6,08E-04 | 3,79E-01 | 20,87158 | HMOX1;USF1;PDK2 | 3 |
| **peroxisome fission (GO:0016559)** | 2/10 | 2,51E-03 | 5,65E-01 | 32,84603 | PEX11A;PEX11B | 3 |
| **mitotic cell cycle (GO:0000278)** | 8/404 | 1,37E-05 | 8,28E-03 | 8,40275 | UBE2C;NEK6;KIF23;KIF2C;CENPA;AURKB;NCAPH;AURKA | 6 |
| **regulation of chromosome segregation (GO:0051983)** | 4/63 | 2,70E-05 | 8,28E-03 | 26,43536 | UBE2C;KIF2C;KNSTRN;AURKB | 6 |
| **mitotic nuclear division (GO:0007067)** | 6/240 | 4,97E-05 | 8,55E-03 | 10,31450 | CDCA3;REEP4;NEK6;KIF2C;AURKB;AURKA | 6 |
| **positive regulation of peptidyl-tyrosine phosphorylation (GO:0050731)** | 3/139 | 8,93E-05 | 2,15E-02 | 43,78897 | FLT3;CCL5;NEURL1 | 5 |
| **positive regulation of protein tyrosine kinase activity (GO:0061098)** | 2/32 | 1,91E-04 | 2,15E-02 | 120,95152 | CCL5;NEURL1 | 5 |
| **regulation of peptidyl-tyrosine phosphorylation (GO:0050730)** | 3/189 | 2,22E-04 | 2,15E-02 | 31,93710 | FLT3;CCL5;NEURL1 | 5 |
| **regulation of ubiquitin-protein transferase activity (GO:0051438)** | 1/103 | 6,01E-02 | 1,50E-01 | 17,72371 | ZER1 | 7 |
| **cell growth (GO:0016049)** | 1/106 | 6,18E-02 | 1,50E-01 | 17,21472 | ATAD3A | 7 |
| **regulation of ligase activity (GO:0051340)** | 1/108 | 6,29E-02 | 1,50E-01 | 16,89125 | ZER1 | 7 |
| **regeneration (GO:0031099)** | 2/97 | 1,39E-04 | 3,19E-03 | 209,48421 | LCP1;TM4SF4 | 10 |
| **wound healing, spreading of cells (GO:0044319)** | 1/15 | 3,00E-03 | 2,92E-02 | 475,76190 | LCP1 | 10 |
| **tissue regeneration (GO:0042246)** | 1/28 | 5,59E-03 | 2,92E-02 | 246,53086 | TM4SF4 | 10 |
| **leukotriene metabolic process (GO:0006691)** | 2/37 | 5,00E-04 | 1,13E-01 | 71,23929 | PLA2G1B;DPEP2 | 4 |
| **fatty acid derivative metabolic process (GO:1901568)** | 2/88 | 2,80E-03 | 1,56E-01 | 28,91860 | PLA2G1B;DPEP2 | 4 |
| **icosanoid metabolic process (GO:0006690)** | 2/88 | 2,80E-03 | 1,56E-01 | 28,91860 | PLA2G1B;DPEP2 | 4 |
| **fucosylation (GO:0036065)** | 3/16 | 1,16E-03 | 8,36E-01 | 17,23776 | FUT6;FUT2;FUT3 | 1 |
| **gamma-aminobutyric acid signaling pathway (GO:0007214)** | 3/24 | 3,87E-03 | 8,36E-01 | 10,66667 | GABRB2;CACNA1A;GABRE | 1 |
| **sensory perception of pain (GO:0019233)** | 4/50 | 4,42E-03 | 8,36E-01 | 6,50918 | SCN10A;CNR2;TAC1;AQP1 | 1 |
| **protein secretion (GO:0009306)** | 4/67 | 2,77E-03 | 6,49E-01 | 7,38114 | ABCA1;PLEK;LTBP2;CBLN1 | 11 |
| **cerebellar granule cell differentiation (GO:0021707)** | 2/10 | 3,23E-03 | 6,49E-01 | 28,80523 | ATP2B2;CBLN1 | 11 |
| **regulation of humoral immune response mediated by circulating immunoglobulin (GO:0002923)** | 2/12 | 4,69E-03 | 6,49E-01 | 23,04186 | HPX;LTA | 11 |
| **cellular defense response (GO:0006968)** | 3/60 | 1,40E-05 | 3,47E-03 | 80,67611 | CD160;C5AR1;LY96 | 8 |
| **inflammatory response (GO:0006954)** | 3/376 | 3,08E-03 | 1,31E-01 | 12,13302 | KCNJ10;C5AR1;LY96 | 8 |
| **immune response-activating signal transduction (GO:0002757)** | 3/440 | 4,78E-03 | 1,31E-01 | 10,32230 | C5AR1;LY96;SLA2 | 8 |
| **artery development (GO:0060840)** | 1/9 | 1,03E-02 | 2,30E-01 | 113,46023 | NKX3-1 | 9 |
| **regulation of epithelial cell proliferation involved in prostate gland development (GO:0060768)** | 1/10 | 1,14E-02 | 2,30E-01 | 100,84848 | NKX3-1 | 9 |
| **positive regulation of steroid hormone secretion (GO:2000833)** | 1/11 | 1,26E-02 | 2,30E-01 | 90,75909 | NKX3-1 | 9 |
